# Application of triazoles in the structural modification of natural products

**DOI:** 10.1080/14756366.2021.1890066

**Published:** 2021-06-24

**Authors:** Hong-Yan Guo, Zheng-Ai Chen, Qing-Kun Shen, Zhe-Shan Quan

**Affiliations:** a Key Laboratory of Natural Medicines of the Changbai Mountain, Affifiliated Ministry of Education, College of Pharmacy, Yanbian University, Affiliated Hospital of Yanbian University, Yanji, Jilin, China; b Department of Pharmacology, Medical School of Yanbian University, Yanji, Jilin, China

**Keywords:** Natural products, triazole, biological activity

## Abstract

Nature products have been extensively used in the discovery and development of new drugs, as the most important source of drugs. The triazole ring is one of main pharmacophore of the nitrogen-containing heterocycles. Thus, a new class of triazole-containing natural product conjugates has been synthesised. These compounds reportedly exert anticancer, anti-inflammatory, antimicrobial, antiparasitic, antiviral, antioxidant, anti-Alzheimer, and enzyme inhibitory effects. This review summarises the research progress of triazole-containing natural product derivatives involved in medicinal chemistry in the past six years. This review provides insights and perspectives that will help scientists in the fields of organic synthesis, medicinal chemistry, phytochemistry, and pharmacology.

## 1. Introduction

More than 200 years ago, a 21-year-old pharmacist’s apprentice named Friedrich Sertürner isolated the first pharmacologically active pure compound from a plant. This compound was morphine derived from opium produced by cut seed pods of the poppy *Papaver somniferum*^1^. This opened an era in which the precise dosage of the purification, research and application of botanicals would not change with the source or age of the substance. After World War II, due to the discovery of penicillin, drug research expanded to large-scale screening of microorganisms to find new antibiotics. By 1990, drugs composed of natural products or analogs inspired by natural products accounted for 80%. The discovery and production of anticancer drugs (e.g. doxorubicin and taxol), immunosuppressants for organ transplants (e.g. rapamycins and cyclosporine), lipid control agents (e.g. lovastatin and analogs), antimalarials (e.g. artemisinin and quinine), antiparasitics (e.g. avermectin), and antibiotics (e.g. tetracycline, penicillin, and erythromycin) revolutionised medicine. It is not difficult to see from these findings that natural products play a very important role in the process of drug discovery and development. Newman’s team published a series of overviews of natural products as sources of new drugs^2–6^, and also reported the sources of antitumor compounds^7^, publishing intermediate reports describing natural products as leads to potential drugs^8^. All of these articles emphasise the inexhaustible importance of natural products and/or natural product structures in drug discovery and development.

**Figure 1.**
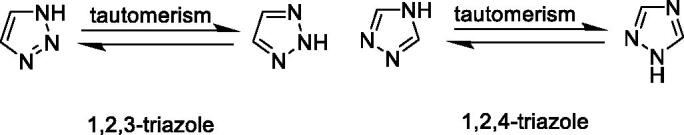
Chemical structures of 1,2,3-triazole and 1,2,4-triazole motifs.

**Figure 2.**
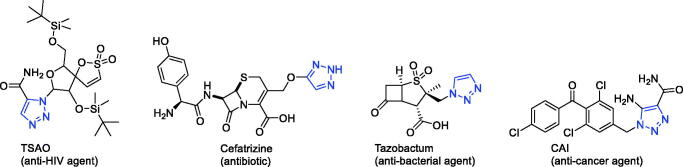
Some of the drugs available in the market containing 1,2,3-triazole ring.

Many marketed drugs contain heterocycles, and triazoles with a five-membered ring composed of two carbon atoms and three nitrogen atoms exist in different heterocycles There are two types of triazole – 1,2,3-triazole and 1,2,4-triazole (Figure 1)^9^. Triazole can be readily obtained, and the framework can act as an amide, ester, carboxylic acid, and other heterocycles such as pyrazole isosteres^10^. By affecting the hydrogen bonding ability, polarity and lipophilicity of the molecules, the triazole moiety can improve the physicochemical properties, toxicology, pharmacokinetics and pharmacology of the compounds^11^^,^^12^. The synthetic moieties containing these molecular structures have been used extensively in the discovery of drugs due to their low occurrence in nature^13^.Meanwhile, on the basis of the literature, triazole and its derivatives have aroused enormous interest owing to their pharmaceutical and therapeutic applications, including their use as anticonvulsant^14–17^, antidepressant^18^, anticancer^19–23^, antiviral^24^, antimicrobial^25–33^, anti-acetylcholinesterase^34^, anti-inflammatory^35^^,^^36^, antioxidant^37–40^, antiparasitic^41–43^, and anti-diabetic drugs^44^. Their ability to produce various non-covalent interactions to improve solubility and binding to bimolecular targets may be the reason for this wide applicability^45^. Furthermore, a number of drugs that contain 1,2,3-triazole scaffolds, including TSAO^46^ (anti-HIV agent), Cefatrizine^47^ (an antibiotic), CAI^48^ (anti-cancer agent), and Tazobactum^49^ (anti-bacterial agent), are currently used in clinical applications (Figure 2). The favourable properties of the enhanced biological activities of the triazole ring include hydrogen bonding capability under *in vivo* conditions, a strong dipole moment, high chemical stability (they are typically inert for oxidising and reducing agents), and rigidity.^33^

Therefore, given that natural products have become the source of most active ingredients in medicines, and the interest focussed on triazole compounds is increasing recently, we will review the latest research progress in regard to triazole-containing natural products in pharmaceutical chemistry.

## 2. Biological activities

### 2.1. Anticancer activity

#### 2.1.1. Anticancer activity of lung cancer

**Figure 3.**
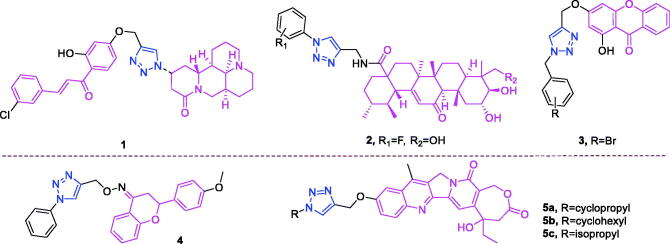
The chemical structures of anti-lung cancer compounds **1–5**.

Matrine (chemical formula: C_15_H_24_N_2_O, molecular weight: 248.36, (7a*S*,13a*R*,13b*R*,13 c*S*)-dodecahydro-1*H*,5*H*,10*H*-dipyrido[2,1-*f*:3′,2′,1′-*ij*][1, 6]naphthyridin-10-one) is a quinolizidine alkaloid that is an important active compound found in the root of *Sophora flavescens* Ait (also known as Kushen). Zhao et al.^50^ found that the conjugation of matrine, 1*H*-1,2,3-triazol, and chalcones could form novel anticancer agents that exerted synergistic effects where the double bond of the α,β-unsaturated moiety plays a dominant role. Adding 2′-OH into the A ring or substituting the B ring of chalcone with EWGs may increase the anticancer activity of matrine–triazole–chalcone conjugates. Among the conjugates, compound 1 (Figure 3) was 8.0-fold more potent (IC_50_ = 5.01 ± 0.59 µM) than was 5-fluorouracil (IC_50_ = 40.38 ± 4.61 µM) and possesses comparable potency to that of paclitaxel (IC_50_ = 2.82 ± 0.31 µM) against A549 cells. Additionally, compound **1** possesses a relatively broad anticancer spectrum and exhibits less cytotoxicity (IC_50_ = 39.21 ± 4.31 µM) than that of 5-fluorouracil (IC_50_ = 22.36 ± 2.09 µM) and paclitaxel (IC_50_ = 20.01 ± 2.38 µM) against NIH3T3 cells. Flow cytometry tests demonstrated that compound **1** could induce apoptosis in A549 cells in a concentration-dependent manner, and efficiently suppressed human tumour growth in a mouse xenograft model without causing obvious toxicities.

Asiatic acid (chemical formula: C_30_H_48_O_5_, molecular weight: 488.70, (2α,3β,4α)-2,3,23-trihydroxyurs-12-en-28-oic acid) is a pentacyclic triterpenoid isolated from the tropical medicinal plant *Centella asiatica* (Apiaceae). Huang et al.^51^ synthesised this compound and discovered that asiatic acid-based 1,2,3-triazoles derivatives could act as antitumor agents by blocking nuclear factor kappa B (NF-κB) activation and cell migration. Among the tested compounds, the hydroxyl group on R_2_ is more beneficial than the acetyl group. Polar groups were investigated at R_1_ using halogen group substitution, where the 2-fluorine derivative with hydroxyl moiety substitution at R_2_ (**2**) (Figure 3), which showed the highest activity among the investigated compounds, yielded in a significantly lower IC_50_ of 0.14 µM. A molecular docking study was performed to identify key interactions between compound **2** and NF-κB, where the 1,2,3-triazoles moiety and the hydroxyl groups of the asiatic acid skeleton were important for improving inhibitory activity. Subsequently, surface plasmon resonance analysis verified the high affinity between compound **2** and NF-κB protein with an equilibrium dissociation constant (KD) value of 0.36 µM. Further studies revealed that compound **2** markedly inhibited NF-κB DNA binding, nuclear translocation, and IκBα phosphorylation. Moreover, in *vitro* antitumor activity screening revealed that compound **2** (IC_50_ = 2.67 ± 0.06 µM) exhibited the best anticancer activity against A549 cells, and this was achieved, at least in part, through the inhibition of NF-κB activity. Treatment of A549 cells with compound **2** resulted in the induction of apoptosis and inhibition of cell migration *in vitro*.

Xanthone (chemical formula: C_13_H_8_O_2_, molecular weight: 196.20, 9*H*-xanthen-9-one) is a bioactive substance that can be isolated from plants and from microorganisms^52^. The basic skeleton of this compound is a biphenyl pyranone possessing a planar three-ring structure. Wu et al.^53^ synthesised several xanthone derivatives and evaluated their cytotoxicity. The IC_50_ analysis indicated that the inhibitory activity of the 18 target compounds was higher than that of the original xanthone intermediate. The extensional structure of various substituted groups resulted in a significantly increased anticancer effect versus compound with the benzyl group. In detail, the compounds with substituted deactivating groups possessed higher activity versus those with electrondonating groups. Of note, the compounds with weak electron-withdrawing groups demonstrated the highest activity in this series. Among compounds with substituted halogen, compound **3** (Figure 3) with replaced para bromide was the most active agent against A549 cancer cells (IC_50_ = 32.4 ± 2.2 µM). Western blotting analyses revealed that compound **3** significantly increased the expression of caspase 3, Bax, and c-Jun N-terminal kinase and also positively regulated p53 in cancer cells.

Flavanone (chemical formula: C_15_H_12_O_2_, molecular weight: 224.25, 2,3-dihydro-2-phenyl-4*H*-1-benzopyran-4-one) is a flavonoid that exists as a polyphenol found in the plant kingdom. On average, the intake of flavonoids is approximately 50–150 mg per day from vegetables, fruits, and other food sources^54^. Flavanone is an important natural secondary metabolite. A series of new flavanone-triazole hybrids were synthesised by Gutam et al.^55^ The entire synthesised group of compounds exhibited a subjective but diverse cytotoxic effect against the HCT-15, HeLa, and NCI-H522 cell lines. Among these compounds, compound **4** (Figure 3) exhibited the highest cytotoxicity against NCI-H522 cells (IC_50_ = 5.4 µM) and possessed an improved safety profile.

Camptothecin (chemical formula: C_20_H_16_N_2_O_4_, molecular weight: 348.35, (4*S*)-4-Ethyl-4-hydroxy-1*H*-pyrano[3′,4′:6,7]indolizino[1,2-*b*]quinoline-3,14(4*H*,12*H*)-dione) is a topoisomerase I inhibitor that was first isolated from *Camptotheca acuminata* by Wall and Wani in 1966^56^. Xu et al.^57^ reported the modifications and SAR of homocamptothecin at position C10 and used these findings to develop potent topoisomerase I inhibitors for anticancer drug discovery. Compounds **5a**, **5b**, and **5c** (Figure 3), possessing cyclopropyl, isopropyl, and cyclohexyl groups, respectively, exhibited very high inhibitory activities that were 4–6 times more effective than camptothecin against A549 cells. The IC_50_ values of these compounds were 30, 30, and 50 nM, respectively, and compound **5a** exhibited stronger Topo I-dependent cytotoxic activity than did camptothecin at concentrations of 100 and 10 µM. Furthermore, compound **6j** could cause cell cycle arrest in the G2 and S phases at a concentration of 0.1 µM. Interestingly, most of the alkyl and cycloalkyl groups promoted the antiproliferative activities of 1,2,3-triazole homocamptothecin derivatives against A549 cells, while the benzyl groups did not. On the contrary, the benzyl group was favourable to MDA-MB-435 cells. Surprisingly, all the glycosyl compounds showed moderate antiproliferative activities against MDA-MB-435 and HCT116, which might be due to their relatively bulky spatial positions. These findings provide valuable insights for further development of more effective homocamptothecins as antitumor agents.

#### 2.1.2. Anticancer activity of breast cancer

**Figure 4.**
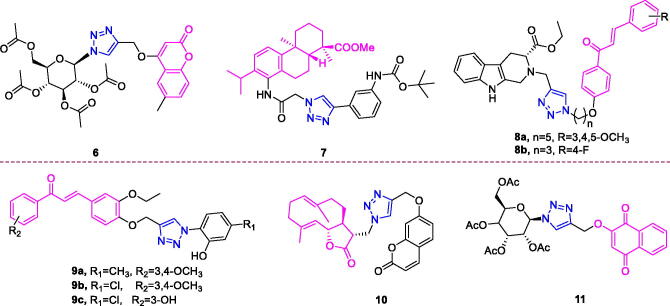
The chemical structures of anti-breast cancer compounds **6–11**.

Coumarin (chemical formula: C_9_H_6_O_2_, molecular weight: 146.14, 2*H*-1-Benzopyran-2-one) was discovered in 1820 as a derivative of the tonka bean. Coumarin exists in many plants in the form of glycosides. Natural and synthetic coumarin derivatives have attracted great attention among medicinal chemists due to of their wide range of biological activities. The anticancer activity of the newly synthesised triazole-linked *N*-glycosides of coumarins and quinolones was determined by Kumari et al.^58^ Compound **6** (Figure 4) displayed low micromolar (IC_50_ = 10.97 µM) and selective toxicity against MCF-7, a breast cancer cell line. Further study revealed that the anticancer activity of the active compound was due to the formation of reactive oxygen species (ROS) however without significant nuclear DNA damage. Apart from causing DNA lesions, ROS production in the cell can also cause oxidative modifications of proteins leading to their altered functions in the cell or leads to lipid peroxidation which can generate toxic products in the cell. Since in study the active compound showed breast cancer cell line (MCF-7) specific cell death without significant nuclear DNA damage, it might be possible that other cellular macromolecules like proteins or lipids essential for the survival of targeted cell lines could be the target of this ROS generation.

Dehydroabietic acid (chemical formula: C_20_H_28_O_2_, molecular weight: 300.44, (1 *R*,4a*S*,10a*R*)-1,2,3,4,4a,9,10,10a-octahydro-1,4a-dimethyl-7-(1-methylethyl)-1-phenanthrenecarboxylic acid) is a natural resin acid that can be easily obtained from disproportionated rosin. A series of novel C-14 1,2,3-triazole-tethered dehydroabietic acid derivatives possess antiproliferative activity^59^. These new analogs remained effective against adriamycin-resistant MCF-7 cells at low concentrations in a dose-dependent manner. The results suggesting that the introduction of 1,2,3-triazole moiety was beneficial to cytotoxicity. Further, the effect of various substituents on the C-4 position of the 1,2,3-triazole moiety was also examined and the structure activity relationship (SAR) studies revealed that the introduction of aromatic substituents was crucial for the potent cytotoxicity. Generally, the introduction of electron-rich aromatic ring system could significantly increase the cytotoxicity. Whereas, the introduction of electron-poor aromatic ring system would hamper the cytotoxicity. For example, pyridyl- and nitrobenzyl-substituted analogues only showed weak or loss of cytotoxicities. In comparison, the saturated alkyl-, ester- and trimethyl chlorosilane- on the C-4 position of the 1,2,3-triazole moiety were only associated with moderate increase in the growth inhibitory effect. In particular, derivative **7** (Figure 4) possessing a 3-(*tert*-butoxycarbonylamino)phenyl-substituted thiazole moiety exhibited the highest potency with IC_50_ values ranging from 0.7 µM to 1.2 µM, and these values are more potent than those of the commercial anticancer drug 5-Fu (average IC_50_ value 16.1 µM). Moreover, compound **7** not only possessed broad-spectrum antiproliferative activities against a series of tumour cells derived from different organs, but also it exhibited very weak cytotoxicity on normal cells, implying the existence of a therapeutic window for the use of compound **7**.

Chalcones (chemical formula: C_15_H_12_O, molecular weight: 280.26, (*E*)-1,3-diphenyl-2-propen-1-one) are naturally occurring flavonoids that possess 1,3-diphenyl-2-propen-1-one as their framework. Chalcone is an α,β-unsaturated ketone that represents a central core for a variety of important bioactive molecules. A series of 1H-1,2,3 triazole-grafted tetrahydro-β-carboline-chalcone/ferrocenylchalcone conjugates was synthesised by Sharma et al.^60^ Analysis of SAR revealed that aryl chalcone based conjugates showed better anti-proliferative activities on both the cell lines tested. Among TH*β*C-chalcone conjugates, the nature of substituent on phenyl ring of chalcone predominantly played an important role in enhancing the cytotoxicity on breast cancer cell lines whereas length of alkyl chain hardly affected the activities. Compounds with electron donating tri-methoxy substituents on phenyl ring displayed appreciable cytotoxicities on breast cancer cells as compared to compounds with mono-methoxy substituent, which were inactive on both breast cancer cell lines. Among trimethoxylated conjugates, Compound **8a** (Figure 4) possessed an electron-donating trimethoxy substituent on the phenyl ring of chalcone and pentyl as a spacer and was the most active against MDA-MB-231 cells with an IC_50_ value of 21.99 µM and was therefore ∼3 folds potent than Tamoxifen. Interestingly, the compounds with electron withdrawing fluorosubstitution at phenyl ring were found to be the most active amongst all the synthesised conjugates. Among these conjugates, compound **8b** (Figure 4), possessing an optimum combination of electron-withdrawing and lipophilic 4-fluoro substituents on the phenyl ring of chalcone and a propyl chain as the spacer, proved to be the most potent with an IC_50_ value of 10.33 µM against MCF-7. Gurrapu et al.^61^ also synthesised novel 1,2,3-triazole chalcone hybrids as potential anticancer agents. All of these compounds were effective**;** however, meta methyl substituent attached to the triazole ring meta, para dimethoxy substituted attached to the chalcone ring of compounds **9a** (Figure 4), meta chloro substitutes attached to the triazole ring and meta, para dimethoxy substituted attached to the chalcone ring of compound **9b** (Figure 4), and meta chloro substituent attached to the triazole ring and meta hydroxy substituted attached to the chalcone ring of compound **9c** (Figure 4) were nearly equipotent and exhibited increased efficiently against cancer cell lines. In particular, **9b** exhibited the best cytotoxic activity against MCF-7 and other cell lines, displaying an IC_50_ of 1.27 µM and 0.02 µM at 24 and 48 h, respectively. The other compounds exhibited intermediate to moderate cytotoxic activities against the tumour cells in comparison to the cytotoxicity of cisplatin. Finally, SAR data revealed that compounds which have chloro and methoxy substituent at different position have shown promising activity when compared with other derivatives and remaining compounds showed moderate cytotoxic activity.

Costunolide (chemical formula: C_15_H_20_O_2_, molecular weight: 232.32, (3a*S*,6*E*,10*E*,11a*R*)-3a,4,5,8,9,11a-hexahydro-6,10-dimethyl-3-methylenecyclodeca[*b*]furan-2(3*H*)-one) is a principal active sesquiterpene lactone derived from *the medicinal plant Aucklandia lappa Decne*^62^. Kumar et al.^63^ successfully synthesised 20 analogs of costunolide and dehydrocostuslactone and evaluated their anticancer activities. It is clear that majority of the derivatives synthesised displayed higher anticancer activity than the parent compounds, costunolide and dehydrocostuslactone against the tested cell lines. Compound **10** (Figure 4) was demonstrated as best analog with a GI_50_ of < 0.12 µM against the MDA MB-231 cell line, a value that is better than that of the parent compound costunolide (GI_50_ = 0.56 µM). These preliminary studies laid a solid foundation for further lead optimisation of this class of compounds by a systematic chemical modification including the synthesis of water-soluble compounds to improve their overall pharmaceutical properties.

Lawsone (chemical formula: C_15_H_20_O_2_, molecular weight: 232.32, 2-Hydroxy-1,4-dihydronaphthalene-1,4-dione) is a natural bioactive compound isolated from plants of the genus *Lawsonia*. Ottoni et al.^64^ synthesised two series of glycosidic derivatives of Lawsone, and these corresponded to classical glycosides and glycosyl triazoles. All compounds displayed acceptable activity against the SKBR-3 cell line with IC_50_ values below 10 µM. The greater activity of peracetylated glycosides and glycosyl triazoles as compared to lawsone is probably due to the more favourable lipophilic–hydrophilic balance that has been achieved with the peracetylated glycosyl derivatives which could be absorbed by tumour cells more easily. The most promising derivative was the glycosyl triazole derived from peracetylated d-glucose (**11**) (Figure 4), exhibiting improved cytotoxicity against SKBR-3 cells (IC_50_ = 0.78 µM) and superior selectivity towards the tumour cell line (SI > 20). All compounds described in this work were more active than was Lawsone, thus indicating the importance of the carbohydrate and glycosyl triazole moiety for activity.

#### 2.1.3. Anticancer activity of gastric cancer

**Figure 5.**
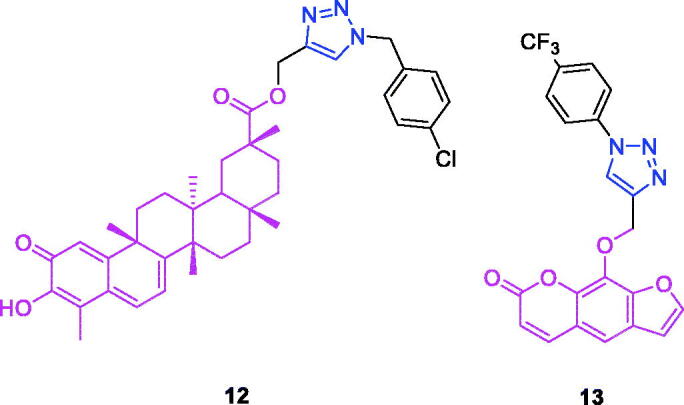
The chemical structures of anti-gastric cancer compounds **12–13**.

Celastrol (Chemical formula: C_29_H_38_O_4_, molecular weight: 450.61, (9β,13α,14β,20α)-3-Hydroxy-9,13-dimethyl-2-oxo-24,25,26-trinoroleana-1(10),3,5,7-tetraen-29-oic acid) is a quinone methide triterpene that is an active ingredient first extracted from the roots of the Chinese medicinal plant “Thunder of God Vine” (Celastraceae, Tripterygium). Three series of novel celastrol derivatives were designed and synthesised by Zhang et al.^65^ The introduction of 1,2,3-triazole linked to benzyl fluorine exerted a minor influence on the anticancer activity of celastrol. However, the celastrol derivative **12** (Figure 5), a 1,2,3-triazole linked to benzyl chloride, exerted an effective anti-proliferative effect on AGS cells (IC_50_ = 0.97 µM).

Xanthotoxin (chemical formula: C_12_H_8_O_4_, molecular weight: 216.19, 9-methoxy-7*H*-furo[3,2-*g*][1]benzopyran-7-one) is a furanocoumarin and an active compound of the traditional Egyptian medicinal plant *Ammi majus L*. Xanthotoxin-triazole derivatives possess antiproliferative properties^66^. The SAR analysis revealed that the phenyl-substituted derivatives showed better activity compared with the benzyl-substituted derivatives and highlighted the importance of the substituted benzene of this molecular modification for the antiproliferative activity in xanthotoxin-linked 1,2,3-triazoles. Among the phenyl-substituted derivatives, the order of potency was p-CF_3_ > p-Cl > p-F > p-CH_3_ > p-OCH_3_. Additionally, for the phenyl-substituted xanthotoxin-linked 1,2,4-triazoles, the order of potency was p-F > p-CF_3_. Thus, compounds with an electron-withdrawing group (–CF_3_, –Cl, –F) at the 4-position of the benzyl moiety displayed better antitumor activity. Among the synthesised compounds, 9-((1-(4-(trifluoromethyl)phenyl)-1*H*-1,2,3-triazol-4-yl)methoxy)-7*H*-furo[3,2-g]chromen-7-one (**13**) (Figure 5) exhibited the strongest antiproliferative activity against AGS cells (IC_50_ = 7.5 µM) and possessed improved activity compared to those of the lead compound (xanthotoxin, IC_50_ > 100 µM) and the reference drug (5-fluorouracil, IC_50_ = 29.6 µM). The IC_50_ value of compound **13** in L02 cells was 13.3-fold higher than that in the AGS cells. Therefore, the compound exhibited improved therapeutic activity and specificity compared to that of the positive control 5-fluorouracil. Cell cycle analysis revealed that compound **13** inhibited cell growth via the induction of S/G2 phase arrest in AGS cells.

#### 2.1.4. Anticancer activity of ovarian cancer

**Figure 6.**
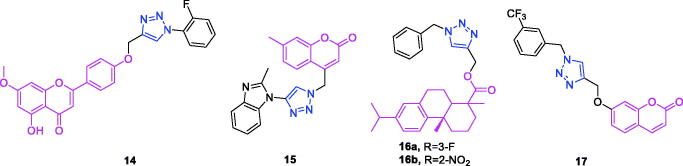
The chemical structures of anti-ovarian cancer compound **14**, anti-liver cancer compounds **15–16**, and anti-pancreatic cancer compound **17**.

Apigenin (chemical formula: C_15_H_10_O_5_, molecular weight: 270.24, 5,7-dihydroxy-2-(4-hydroxyphenyl)-4*H*-1-benzopyran-4-one) is a naturally occurring flavonoid compound that exists in a variety of plants in the form of phyto-yellow pigment, and this compound is primarily derived from the Umbelliferae plant *Apium graveolens L*. Qi et al.^67^ found that novel triazole analogs of apigenin-7-methyl ether exhibit potent antitumor activity against ovarian carcinoma cells via the induction of mitochondria-mediated apoptosis. Of all the derivatives, the derivative **14** (Figure 6) exhibited significant and dose-dependent anticancer activity against the SKOV3 ovarian cancer cell line. The IC_50_ of compound **14** was 10 µM against the SKOV3 cancer cell line. Compound **14** induced apoptosis in SKOV3 cancer cells through the accretion of reactive oxygen species and a reduction in mitochondrial membrane potential. This molecule also modulated the expression of B-cell lymphoma 2 (Bcl-2) and Bcl-2-associated X protein.

#### 2.1.5. Anticancer activity of liver cancer

As noted earlier, derivatives of coumarin-triazole **15** exhibit anti-breast cancer activity^58^. Kraljevic et al.^68^ synthesised a new 4-substituted 1,2,3-triazole-coumarin hybrid possessing anti-liver cancer activity. The incorporation of benzofused heterocycles led to improvements in activities. The 7-methylcoumarin-1,2,3-triazole-2-methylbenzimidazole hybrid **15** (Figure 6) can be highlighted as exerting the highest cytotoxicity against hepatocellular carcinoma HepG2 cells with an IC_50_ value of 0.9 µM and high selectivity (SI = 50). This compound induced cell death that occurred primarily due to early apoptosis. The growth-suppressive properties of compound **15** in HepG2 cells could be associated with its ability to inhibit 5-lipoxygenase (5-LO) and acid ceramidase activities, as these inhibitions may, in turn, lead to the accumulation of arachidonic acid and ceramide, respectively.

Dehydroabietic acid-triazole derivatives not only exhibit anti-breast cancer activity^59^ but also possess anti-liver cancer activity^69^. A series of dehydroabietic acid-coupled 1,2,3-triazole derivatives was synthesised by Li et al.^69^ The synthesised compounds were screened for cytotoxic activity against a panel of four human cancer cell lines and the human HL-7702 normal cell line using a 3-(4,5-dimethylthiazolyl-2)-2,5-diphenyltetrazoliumbromide] (MTT) assay. Of these compounds, a number exhibited better anticancer activity against the tested cancer cell lines compared to that of the positive control cisplatin, and they also exhibited low cytotoxicity against the human normal liver cell line HL-7702, indicating that the introduction of 1,2,3-triazole moiety on the DHAA skeleton increased anti-tumour activity. In particular, compounds **16a** and **16b** (Figure 6) exhibited good antitumor activity against HepG2 with IC_50_ values of 5.90 ± 0.41 and 6.25 ± 0.37 µM, better than those of positive control cisplatin.

#### 2.1.6. Anticancer activity of pancreatic cancer

Coumarin-triazole derivatives not only possess anti-breast cancer activity^58^ but also exhibit anti-liver cancer activity^68^ and anti-pancreatic cancer activity^70^. Farley et al.^70^ synthesised a series of functionalised coumarins and evaluated their capacity to inhibit the resistance to starvation in pancreatic cancer cells. Evaluation of two trifluoromethylphenyl compounds against three cancer cell lines showed that position of the trifluoromethyl substituent on the phenyl ring of these compounds (*meta-* vs *para-*) was correlated to selectivity for activity against MIA PaCa-2 cell line. This relatively small change in structure had a substantial effect on activity. The *meta*-trifluoromethylphenyl derivative **17** (Figure 6) exhibited preferential cytotoxicity against PANC-1, Capan-1, and MIA PaCa-2 cells with PC_50_ concentrations of 29, 8.5, and 18 µM, respectively. Apoptosis was determined as the mechanism of cell death (PANC-1, compound **17**), based on a modified ethidium bromide and acridine orange (EB/AO) staining assay.

#### 2.1.7. Anticancer activity of Colon cancer

**Figure 7.**
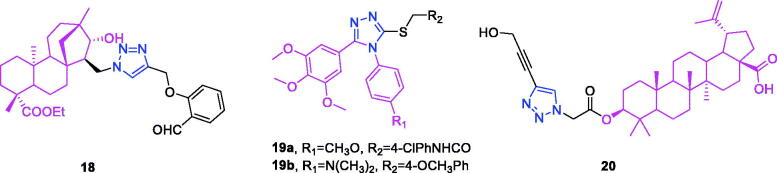
The chemical structures of anti-colon cancer compounds **18–20**.

Isosteviol (chemical formula: C_20_H_30_O_3_, molecular weight: 318.45, (4α,8β,13β)-13-Methyl-16-oxo-17-norkauran-18-oic acid) is a tetracyclic diterpenoid possessing an *ent*-beyerane skeleton that exhibits multifarious bioactivities and can be readily obtained as a metabolite of stevioside isolated from the leaves of the natural *stevia* plant^71^^,^^72^. Liu et al.^73^ designed and synthesised a series of novel 1,2,3-triazole-linked isosteviol derivatives using the Huisgenclick reaction. The cytotoxicities of these compounds against HCT-116 and JEKO-1 cells were screened *in vitro*. From the observed cytotoxic activity data, it has been noticed that all the derivatives of isosteviol showed better cytotoxic activities than their corresponding precursor. More importantly, the inhibitory activities of most compounds were markedly improved as the 1,2,3-triazole subunit was introduced onto the skeleton of isosteviol, which indicated the 1,2,3-triazole fragment exactly played a significant role in inhibiting cancer cell proliferation. Going even further, substituted groups and positions on aromatic ring had a significant effect on cytotoxic activities. Ortho-position on aromatic ring has an important effect on cytotoxic activity. Compounds with aldehyde group on aromatic ring exhibited better inhibitory activities than the unsubstituted compounds. Oxidation of aldehyde group caused weaker activities to the cancer cell lines. To be noteworthiness, the inhibitory activities of isosteviol simultaneously fusing hydroxyl and 1,2,3-triazole subunits were better than that of compounds with only 1,2,3-triazole subunit, which illustrates that introduction of hydroxyl group can result in higher inhibitory activity against HCT-116 cells. In particular, compound **18** (Figure 7) exhibited the most potent inhibitory activity against HCT-116 cells with an IC_50_ value of 2.987 ± 0.098 µM, and this was better than that (3.906 ± 0.261 µM) of the positive control cisplatin. On the basis of these bioactivity data, hologram quantitative structure activity relationship was performed, and a statistically reliable model with good predictive power *(r*^2^ = 0.848, *q*^2^ = 0.544 and *R*^2^_pred_ = 0.982) was achieved. The contribution maps derived from the optimal model explained the individual atomic contributions to the activity for each molecule.

Combretastatin A-4 (chemical formula: C_18_H_20_O_5_, molecular weight: 316.35, 2-methoxy-5-[(1*Z*)-2-(3,4,5-trimethoxyphenyl)ethenyl]phenol) is among the most well-known anticancer agents and was originally isolated from the South African bush willow tree *Combretum caffrum Kuntze* (Combretaceae)^74^^,^^75^. A series of novel alkylsulfanyl-1,2,4-triazoles modified as *cis*-restricted combretastatin A-4 analogs exhibited significant antiproliferative activities^76^. Among the 4-methoxy analogues, displayed that the alkylsulfanyl moiety and its substitutions were critical for keeping antiproliferative effect and the antiproliferative activities were almost lost when the thiol group was free or replaced by methylthio or ethylthio. Secondly, when changing the thiol group of the 3-position of triazole ring with benzylthio, the cytotoxic activities against HepG2, PC-3 and HCT116 cells were significantly increased by the chain elongation. Meanwhile, the introduction of electron withdrawing groups such as fluoro atom on the benzyl group, also caused a slight enhancement of the antiproliferative activity. These results suggest that electronic effect of substituents on benzyl group plays a crucial role on antitumor activities. Besides, linker-length of alkylsulfanyl moiety has also profound effects on the antiproliferative activities. Introduction of phenyl acetylthio substitutes on the 3-position of triazole ring leads to dramatical enhancement of antiproliferative activities against HepG2 cell lines, but naphthyl, cyclopropyl and ethoxyl groups result in dramatical decrease of the activities. It is worth noting that compound **19a** (Figure 7), with N-4 chlorophenyl acetamidethio substitute, showed more potent *in vitro* cytotoxic activities against PC-3 with IC_50_ values of 6.29 µM, which represented threefold improvement in activity compared to combretastatin A-4. Moreover, further flow-activated cell sorting analysis revealed that compound **19a** displayed a significant effect on G_2_/M cell-cycle arrest in a dose dependent manner in PC-3 cells. Within the series of N,N-dimethyl analogues, the effects of substituents on the antiproliferative activities were strongly correlated with the 4-methoxy analogues. Meanwhile, analogue **19b** (Figure 7) was an exception, which displayed fivefold improvement compared to combretastatin A-4 in inhibiting HCT116 cell proliferation with IC_50_ values of 1.15 µM. More interestingly, analog **19b** also displayed the most potent anti-tubulin activity with a percentage of 49% at 10 µM.

Betulinic acid (chemical formula: C_30_H_48_O_3_, molecular weight: 456.7, (3β)-3-hydroxylup-20(29)-en-28-oic acid) is a bioactive pentacyclic lupane-type triterpenoid that can be directly isolated from *Platanus orientalis* stem bark and from many other plants such as the birch tree *Betula spp*. (Betulaceae), *Ziziphus spp*. (Rhamnaceae), *Syzygium spp*. (Myrteceae), *Diospyros spp*. (Ebenaceae), and *Paeonia spp.* (Paeoniaceae)^77^^,^^78^. A new library of compounds possessing a 1,2,3-triazole moiety attached to C-3 of betulinic acid was synthesised, and the anti-cancer activities of these compounds were evaluated (*in vitro)* against different cancer cell lines (i.e. breast, colon, liver, and leukemic) by Chakraborty et al.^79^ The structure activity relationship studies indicate that the 1,2,3-triazole moiety favours the activity when substituted at C-4 (of the heterocycle) with a hydroxymethyl group, but disfavours it when the substituent is an aromatic or heteroaromatic moiety. Compound **20** [(3*S*)-3-{2–(4-(hydroxymethyl-1*H*-1,2,3-triazol-1-yl)acetyloxy}-lup-20(29)-en-28-oic acid] (Figure 7) was found to be the most potent inhibitor of the cell line HT-29 with an IC_50_ value of 14.9 µM. This activity profile was improved compared to that of the parent compound (betulinic acid). Its role as an inducer of apoptosis was investigated in this cell line using an Annexin-V/PI binding assay and by following its capability for ROS generation, depolarisation of mitochondrial transmembrane potential, activation of caspases, PARP cleavage, nuclear degradation, and expression of pro- and anti-apoptotic proteins. This compound exhibited much higher cytotoxicity than did the standard drug 5-fluorouracil; however, it exhibited negligible cytotoxicity towards normal PBMCs. Elevated levels of ROS generation, activation of caspase 3 and caspase 9, DNA fragmentation, higher expression of Bax and Bad, lower expression of Bcl2 and Bcl-xl, and increased levels of Bax/Bcl-xl ratio identified compound **20** as a promising inducer of apoptosis that follows a mitochondria-dependent pathway. Bio-physical studies indicate that compound **20** acts as a minor groove binder to the DNA.

#### 2.1.8. Anti-leukaemia activity

**Figure 8.**
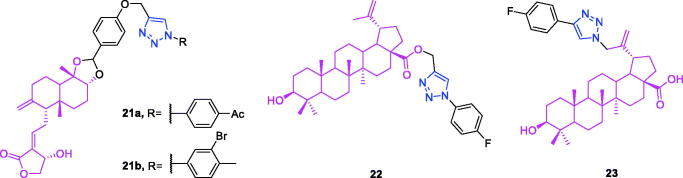
The chemical structures of anti-leukaemia compounds **21–23**.

Andrographolide (chemical formula: C_20_H_30_O_5_, molecular weight: 350.45, (3*E*,4*S*)-3-[2-[(1*R*,4a*S*,5*R*,6*R*,8a*S*)-decahydro-6-hydroxy-5-(hydroxymethyl)-5,8a-dimethyl-2-methylene-1-naphthalenyl]ethylidene]dihydro-4-hydroxy-2(3*H*)-furanone) is one of the labdane diterpenoids that are the principal active constituents of *Andrographis paniculata*. A series of new andrographolide-1,2,3-triazole derivatives were synthesised from the natural bioactive labane-type diterpenoid andrographolide^80^. All of the derivatives were screened against the human cancer cell lines MCF7, MDA-MB-231, COLO205, HepG2, K562, Hela, and HEK293 to evaluate their cytotoxic activity. All of these compounds exhibited anticancer activity selectively against the K562 cell line with IC_50_ values ranging from 8.00 to 17.11 µM and were inactive against the rest of the cell lines. Andrographolide-1,2,3-triazole can be considered as parent moiety, and the substitution at the first position of triazole was varied. It is very clear that the first position with a substituted benzene ring found to be active over unsubstituted or aliphatic substitution, and electron-donating groups are preferred over withdrawing groups. The increase in the carbon chain length linking the substituted benzene with the triazole group reduces the activity. Compounds **21a** and **21b** (Figure 8) exhibited better cytotoxicity against K562 cell lines compared to that of the other compounds in the series with 62.9% and 51.8% inhibition at 50-µM concentration IC_50_ values of 8 and 9.7 µM, respectively.

Betulinic acid-triazole derivatives not only possess anti-colon cancer activity^79^ but also exhibit an anti-leukaemia effect^81^^,^^82^. A novel triazole derivative of betulinic acid induces extrinsic and intrinsic apoptosis in human leukaemia HL-60 cells^81^. The structure activity relationship of structural modifications can be summarised as follow: the more electronegative *p*-fluoro exhibited much better cytotoxicity in all the cancer cell lines compared to *p*-bromo derivative which showed only 61% and 54% of growth inhibition against MiaPaCa-2 and A549 cancer cell lines. The probable reason for the same may be the high lipophilicity, by molecule can penetrate easily to the cells and exerted its activity. Generally, compounds containing electron withdrawing functional groups (–F, –Cl) exhibited more potent cytotoxic effects against the cancer cells compared to the electron donor functional groups (–CH_3_, –OCH_3_). Among all of the tested compounds, compound **22** (Figure 8) displayed promising antiproliferative activity in all four cell lines (HL-60, MIAPaCa2, PC-3, and A549), with IC_50_ values of 7, 5, 7, and 7 µM, respectively. The cytotoxic profile of compound **22** was similar to that of betulinic acid. Based on this, Khan et al. chose this compound for use in further cell death mechanistic studies in HL-60 cells. The effect of compound **22** on DNA damage and apoptosis was investigated using cell cycle analysis. It was observed that treatments with up to 10 µM of compound **22** resulted in mild induction of apoptosis (7%). Furthermore, the cell cycle G1 phase was significantly blocked by compound **22** at a concentration of 20 µM, indicating that it caused a delay in the cell cycle. Compound **22** induced loss of mitochondrial membrane potential in a concentration-dependent manner. Compound **22** at 5 µM induced a 7% loss of mitochondrial membrane potential, and this increased to 18% and 27% at 10 and 20 µM concentrations, respectively. Compound **22** decreased the expression of mitochondria-associated anti-apoptotic protein Bcl-2 in a concentration-dependent manner. HL-60 cells treated with compound **22** exhibited significant loss of mitochondrial membrane potential. Compound **22** significantly decreased the level of the mitochondrial antiapoptotic protein Bcl-2 and increased the expression of the pro-apoptotic protein Bax with a concurrent decrease in the Bcl-2/Bax ratio. Compound **22** treatment results in the activation of caspases and in PARP-1 cleavage. Compound **22** inhibited both Procaspase-9 and Procaspase-8, indicating that it induced apoptosis via both the extrinsic and intrinsic pathways. Therefore, we can conclude that it induces apoptosis via both intrinsic and extrinsic activation pathways in HL-60 cells. The 18 C-30 triazole-substituted betulin and betulinic acid derivatives were synthesised by Shi et al.^82^ These compounds were tested for their cytotoxic activity against the leukaemia cell line HL-60 using an MTT assay. New C-30 triazole-substituted betulinic acid derivatives exhibited improved cytotoxic activity compared to that of betulin derivatives (> 25 µM). The majority of the new triazole-substituted betulinic acid derivatives displayed improved bioactivity compared to that of betulinic acid (11.5 µM). The rank order of potency based on the C-30 triazole substituent is 4-fluorophenyl > *n*-hexyl > phenyl = 2-thienyl > *n*-butyl > cyclopropyl. Overall, the compound **23** (Figure 8) [4-(4-fluorophenyl)-1*H*-1,2,3-triazol-1-yl] betulinic acid possessed the best IC_50_ value (1.3 µM) against the leukaemia cell line HL-60 (eight- to ninefold higher potency than that of betulinic acid). Therefore, larger C-30 side chains with aromatic substitutions were favoured for the cytotoxic activity.

#### 2.1.9. Miscellaneous

**Figure 9.**
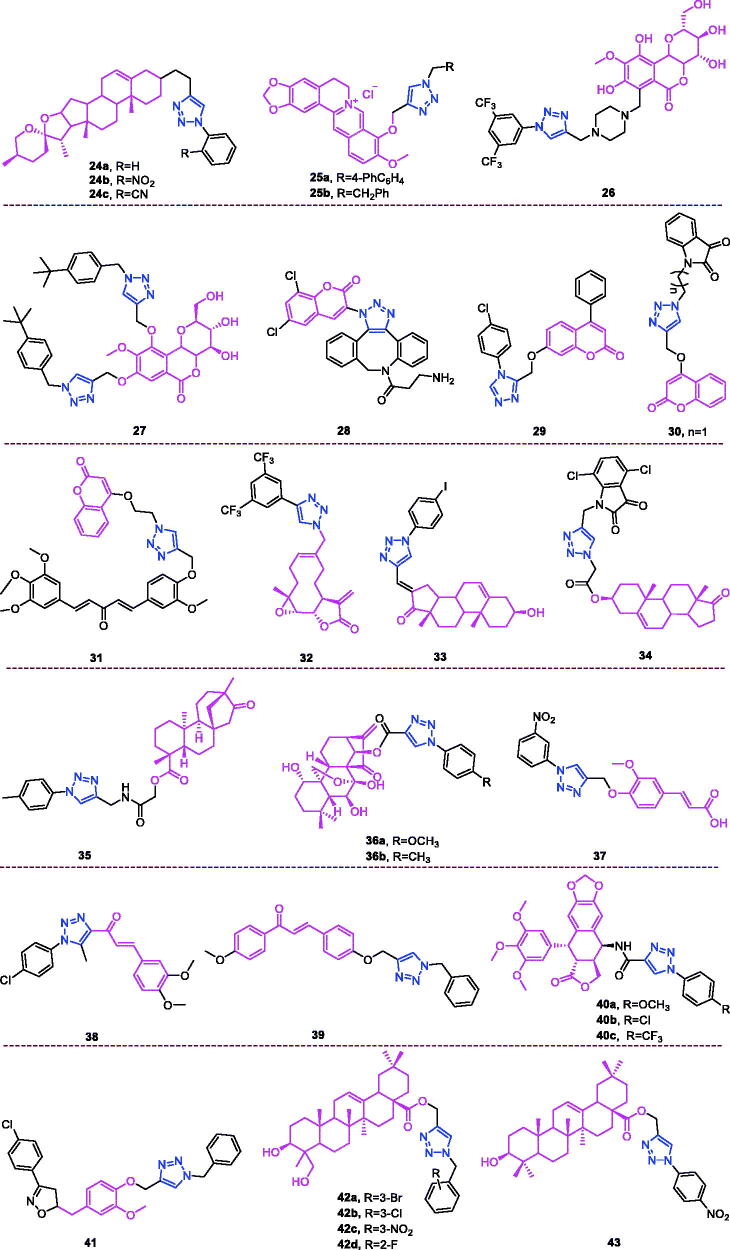
The chemical structures of anti-multiple cancer cells compounds **24–43**.

Diosgenin (chemical formula: C_27_H_42_O_3_, molecular weight: 414.63, (3β,25*S*)-Spirost-5-en-3-ol) is rich in the tubers of *D. deltoidei*, where it exist as a steroidal sapogenin. Diosgenin is structurally similar to cholesterol and to other steroids, and this compound is in high demand in the pharmaceutical industry^83^. Both diosgenin and its analogs exhibited interesting anti-proliferative effects against four human cancer cell lines^84^ (HBL-100 [breast], A549 [lung], HT-29 [colon], and HCT-116 [colon]) according to the results of MTT assays. Among the synthesised analogs, compound **24a** (Figure 9) that possesses a simple phenyl R moiety attached via triazole to the parent molecule was identified as the most potent analog against the A549 cancer cell line. This analog possessed an IC_50_ of 5.54 µM, which was improved compared to that of the positive control (BEZ-235). Compounds **24b** (Figure 9) and **24c** (Figure 9) that possess *o*-nitrophenyl and *o*-cyanophenyl R moieties, respectively, displayed impressive anti-proliferative activity against all the tested human cancer cell lines and exhibited IC_50_ values ranging from 5.77 to 9.44 µM. These observations highlight the beneficial impact of electron withdrawing ortho substituents attached to R moiety towards the anti-proliferative activity.

Berberine (chemical formula: C_20_H_18_NO_4_, molecular weight: 336.37, 5,6-dihydro-9,10-dimethoxybenzo[*g*]-1,3-benzodioxolo[5,6-a]quinolizinium) is a naturally occurring isoquinoline alkaloid that can be isolated from various Berberis plants^85^^,^^86^. Jin et al.^87^ designed and synthesised a series of new triazolyl berberine derivatives. These synthesised compounds and their anticancer activities were evaluated against a panel of four human cancer cell lines that included MCF-7 (breast), MCF-7/ADR (breast), SW-1990 (pancreatic), and SMMC-7721 (liver) and against the non-cancer cell line HUVEC (human umbilical vein endothelial cell). The results revealed that the majority of the compounds exhibited improved anticancer activities against MCF-7 and SMMC-7721 compared to that of berberine. SAR analysis indicated the following: (i) heterocycle substituents at the R position showed poor anticancer activity against four cancer cell lines; (ii) when methylene-naphthalene was substituted for methylene-quinoline, the cytotoxicity was significantly reduced. The results of the *in vitro* anticancer activity tests indicated that compounds **25a** and **25b** (Figure 9) exhibited the most potent inhibitory activities against the SMMC-7721 and SW-1990 cell lines with IC_50_ values of 14.861 ± 2.4 µM and 16.798 ± 3.4 µM, respectively.

Bergenin (chemical formula: C_14_H_16_O_9_, molecular weight: 328.27, (2 *R*,3*S*,4*S*,4a*R*,10b*S*)-3,4,4a,10b-tetrahydro-3,4,8,10-tetrahydroxy-2-(hydroxymethyl)-9-methoxypyrano[3,2-c][2]benzopyran-6(2*H*)-one) represents a dihydroisocoumarin derivative. Kumar et al.^88^ designed and synthesised a series of novel bergenin-trizole hybrid derivatives and evaluated their anti-cancer activities against DU-145, A549, HCT-116, Hep G2, and HeLa cell lines. It is evident that majority of synthetic derivatives displayed better cytotoxic activity than parent compound on A 549, HepG2 and HeLa cell lines. Preliminary structure activity relationship indicates that presence of a substituent such as a chlorine atom or a cyano, floro and CF_3_ group on aromatic triazole partner enhanced activity with IC_50_ values ranging from 1.33 to 9.9 µM on A459 cell as well as HeLa cell lines within the studied series. Among them, compound **26** (Figure 9) demonstrated potent activity against A-549 and HeLa cell lines with IC_50_ values of 1.86 µM and 1.33 µM, respectively, and was equipotent to doxorubicin. Furthermore, mechanistic studies revealed that compound **26** triggered cell cycle arrest at the G_2_/M phase and induced cell apoptosis in a dose- and time-dependent manner. Taken together, these results indicate that compound **26** effectively inhibited tubulin polymerisation and disrupted the intracellular tubulin-microtubule balance, ultimately resulting in prolonged G_2_/M cell cycle arrest. Docking studies also indicated a strong hydrophobic interaction with tubulin, thus leading to stable binding and subsequent apoptosis of cancer cells. A series of triazole derivatives of bergenin were synthesised by Yang et al.^89^ The introduction of 1,2,3-triazole into the bergenin skeleton, exhibit enhancing effect in terms of anti-tumour activities than bergenin. The electron properties and steric effects of the substitutes of the benzene ring affect the antiproliferative activity. Either an electron-donating substituent such as 4-methyl, 4-ethyl and 4-*tert* butyl group or an electron-withdrawing substituent such as 4-methoxy, 4-fluoro, 4-nitrile and 4-nitro group of the benzene ring of the side chain are all more effective than the unsubstituted benzene derivative. Particularly, the introduction of larger steric hindrance substituent on the benzene ring, such as the *tert*-butyl group, caused compound **27** (Figure 9) to exhibit significantly improved antiproliferative activity against three cancer cell lines (EC9706, MGC803, and B16) compared to that of bergenin. The IC_50_ dropped to 6.2 µmol/L, 12.0 µmol/L, and 17.6 µmol/L, respectively, and this compound had a more effective antiproliferative activity than the positive drug 5-fluorouridine against the cancer cell line EC9706.

Coumarin-triazole derivatives not only exhibit anti-breast cancer activity^58^, anti-liver cancer activity^68^, and anti-pancreatic cancer activity^70^, but also they possess activity against multiple types of cancer cells. A series of coumarin-based target-specific probes for cancer theranostic applications that played a dual role in the field of both diagnosis and therapy was screened for anticancer activity against breast cancer (MCF7) and human epitheloid cervix carcinoma (HeLa) cell lines^90^. All hybrids with IC_50_ values ranging from 9.83 to 26.21 µM exhibited high potency against MCF7 and HeLa cell lines, and this potency was comparable to that of cisplatin (IC_50_: 18 and 10 µM) but was less than that of doxorubicin (IC_50_: 5.2 and 3.83 µM). The SAR revealed that the electron-withdrawing groups-Cl and-Br on the 1,2,3-triazole motif boost the anticancer profile in both cell lines, while the electron-donating groups-OH and -OMe and the alkyl groups were unfavourable in regard to activity. The most active was hybrid **28** (Figure 9) (IC_50_: 17.5 and 9.83 µM), and this compound was slightly more potent than was cisplatin and also exhibited the lowest cytotoxicity in normal human foetal lung fibroblast (MRC-5) cells (IC_50_: 185.22 µM). Moreover, hybrid **28** exhibited strong cellular uptake in the MCF-7 cell line. Thus, hybrid **28** may be useful for cancer theranostics due to its high potency, low cytotoxicity, and strong cellular uptake. The coumarin derivatives also exhibited improved antiproliferative activities against several cancer cell lines^91^. It is necessary to point out that the cytotoxic activities of the derivatives with different halogen substitutions on the benzene ring were in the following order, *p*-Cl > *o*-Cl > *m*-Cl, *p*-CH_3_ > *o*-CH_3_ > *m*-CH_3_ and 2.4-(CH_3_)_2_ > 2.6-(CH_3_)_2_. Based on an overall comparison, the compounds derived from structures with electron-withdrawing substituents on the 1,2,4-triazole ring exhibited potent activity, and those with electron-donating substituents on the 1,2,4-triazole ring exhibited moderate activity, against the six cancer cell lines. Compound **29** (Figure 9) exhibited potent activity against AGS, MGC-803, and HCT-116 cell lines, where the IC_50_ values were 2.63 ± 0.17, 3.05 ± 0.29, and 11.57 ± 0.53 µM, respectively. This compound also exerted strong activity against the HeLa cell line, with an IC_50_ value of 13.62 ± 0.86 µM. A more detailed mechanistic study demonstrated that compound **29** could inhibit the proliferation of AGS cancer cells by inducing apoptosis and arresting cells in the G_2_/M phase. In an attempt to develop potent anti-tubulin agents against various cancers, a library of 28 novel triazole-tethered isatin-coumarin hybrids was synthesised through the use of a click chemistry approach^92^. Cytotoxicity results revealed an interesting structure activity relationship for the designed hybrids: (i) type of substituent on isatin and the length of carbon-bridge connecting isatin moiety with triazole ring considerably influences cytotoxic potential of hybrids; (ii) presence of unsubstitued isatin remarkably enhances the cytotoxic potential; (ii) enhanced cytotoxicity for hybrids having isatin with electron withdrawing substituent in comparison to electron donating substituent; (iii) cytotoxicity increases with increase in electronegativity of substituent on isatin. Thus, the overall preference order of R is as follows: H > F > Cl > Br > I > NO_2_ > OCH_3_; (iv) cytotoxicity decreases significantly with the increase in chain length of carbon-bridge. Thus, the overall preference order of n (chain length) is as follows: 1 > 2 > 3 > 4. The most active was hybrid **30** (IC_50_: 0.73, 3.45, and 3.04 µM against THP-1, COLO-205, and HCT-116 cancer cell lines), and this compound also displayed the most potent anti-tubulin activity with an IC_50_ value of 1.06 µM. The *in vitro* tubulin polymerisation assay clearly indicated that these hybrids exert their anticancer activity through tubulin inhibition. Singh et al.^93^ synthesised triazole ring-binding molecule hybrids of C5-curcuminoid and coumarin. Cytotoxicity results revealed an interesting structure activity relationship for these designed hybrids: (i) methoxy substituted phenyl ring remarkably enhances the cytotoxic potential; (ii) placement of a heteroaryl ring such as furan and thiophene in place of the unsubstituted phenyl ring improved the activity profile; (iii) an enhanced effect was observed with the increased number of methoxy substituents on phenyl ring such as trimethoxy phenyl > dimethoxy phenyl > monomethoxy phenyl; (iv) placement of naphthyl ring behaved as a surrogate for dimethoxy substituted phenyl ring; (v) cytotoxicity of hybrids with monomethoxy substituted phenyl ring was found similar to the heteroaryl ring substituted hybrids. Thus, the overall preference order of ring is established as follows: trimethoxy phenyl > dimethoxy phenyl = naphthyl > monomethoxy phenyl = furan = thiophene > phenyl. The most active hybrid **31** with trimethoxy phenyl ring exhibited significant cytotoxicity with IC_50_ values ranging from 0.82 to 4.68 µM against THP-1, HCT-116, and COLO-205 cell lines, respectively. Compound **31** also displayed the most potent anti-tubulin activity, with an IC_50_ value of 1.55 µM.

Melampomagnolide B (chemical formula: C_15_H_20_O_4_, molecular weight: 264.32, (1a*R*,4*E*,7a*S*,10a*S*,10b*R*)-2,3,6,7,7a,8,10a, 10b-Octahydro-5-(hydroxymethyl)-1a-methyl-8-methyleneoxireno^9^^,^^10^ cyclodeca[1,2-b]furan-9(1a*H*)-one) is a sesquiterpene lactone compound extracted from *Magnolia grandiflora*^94^. Melampomagnolide B triazole analogs are potent NF-B inhibitors and anti-cancer agents against both hematological and solid tumour cells^95^. Among them, compound **32** (Figure 9) exhibited promising anticancer activity against cell lines derived from colon cancer, melanoma, renal cancer, and breast cancer sub-panels, and this compound was a particularly potent anticancer agent (GI_50_ = 20 nm) against the RXF 393 renal cancer cell line. Importantly, compound **32** possessed nanomolar activity (EC_50_ = 400–700 nm) against both M9ENL1 and primary AML cell lines. Compound **32** was significantly more potent than was parthenolide as an inhibitor of p65 phosphorylation in both hematological and solid tumour cell lines, indicating its ability to inhibit the NF-κB pathway. In TMD-231 breast cancer cells, treatment with compound **32** reduced the DNA-binding activity of NF-κB through inhibition of IKK-β-mediated p65 phosphorylation and caused elevation of basal IkBa levels through inhibition of constitutive IkBa turnover and NF-κB activation. Molecular docking and dynamic modelling studies indicated that compound **32** interacts with the kinase domain of the monomeric IKKβ subunit, ultimately leading to inhibition of IKKβ activation and compromising phosphorylation of downstream targets in the NF-κB pathway. Dynamic modelling studies revealed that this interaction also causes unwinding of the α-helix of the NEMO binding site on IKKβ.

Dehydroepiandrosterone (chemical formula: C_19_H_28_O_2_, molecular weight: 288.42, (3β)-3-Hydroxyandrost-5-en-17-one) is a unique active substance found in sweet potato and yam. Dehydroepiandrosterone derivatives contain triazole at the C_16_ position and exhibit antiproliferative effects^96^. Based on an overall comparison, the compounds derived from structures with electron-withdrawing substituents on the 1,2,3-triazole ring exhibited potent activity, whereas those with electron-donating substituents on the 1,2,3-triazole ring displayed no apparent activity against the six cancer cell lines. For the 3-substituent compounds, only 3-F substitution exhibited potent activity, and all 2-substituent compounds showed no significant activity. The special 3,4-Cl_2_ replacement exhibited potent activity. The most promising compound was (*E*)-3-hydroxy-16-((1–(4-iodophenyl)-1*H*-1,2,3-triazole-4-yl)methylene)-10,13-dimet-hyl-1,3,4,7,8,9,10,11,12,13,15,16-dodecahydro-2*H*-cyclopenta[a]phenanthren-17(14)-one (compound **33**) (Figure 9), as it exhibited considerably high antiproliferative activity in the HepG-2 cell line with an IC_50_ value of 9.10 µM and considerably high activity against the MCF-7 cell line with an IC_50_ value of 9.18 µM. Flow cytometry assays demonstrated that compound **33** exerted antiproliferative effects by arresting cells in the G2 phase of the cell cycle and by inducing apoptosis. Yu et al.^97^ also used molecular hybridisation methods to efficiently synthesise new anti-proliferative steroid hybrids. These hybrids displayed different antiproliferative activities against various cancer cell lines, with IC_50_ values ranging from 4.06 to > 128 µM. And conclude that 7-chloro or 4, 7-dichloro substituent is beneficial for improving the activity, at least against SH-SY5Y cells. In particular, compound **34** (Figure 9), possessing the 4, 7-dichloro group, exhibited the highest antiproliferative activity with an IC_50_ value of 4.06 µM, comparable to that of 5-fluorouracil (IC_50_ = 3.26 µM) against SH-SY5Y cells. Compound **34** exerted moderate inhibitory activity against MCF-7, U87, MGC-803, and EC109 cells (IC_50_ = 32.25, 9.57, 5.95, and 20.77 µM, respectively). Compound **34** arrested the cell cycle at the G2/M phase, induced apoptosis accompanied by a decrease in mitochondrial membrane potential, and potently inhibited LSD1 (IC_50_ = 3.18 µM). Docking studies indicated that compound **34** formed interactions with the surrounding amino acid residues, and the steroid nucleus occupied the tubular hydrophobic cavity of the active site.

Derivatives of isosteviol-triazole not only possess anti-colon cancer activity^73^, but also they exhibit anti-multiple cancer cell activity^98^. Compounds with different phenyl 1,2,3-triazole chloroacetamide showed considerably higher antiproliferative activity against the HCT-116 and HepG2 cell lines. Perhaps the triazole acts as a hydrogen bond acceptor and binds to some key enzymes involved in cancer cell metabolism, inhibiting their expression. In particular, compound **35** (Figure 9) that possesses a methyl group introduced at the *para* position of the benzene ring exhibited the strongest antiproliferative activity among all of the target compounds (IC_50_ values of 5.38 ± 0.26 µM, 15.91 ± 0.41 µM, and 8.92 ± 0.44 µM against HCT-116, BEL-7402, and HepG2 cell lines, respectively). Compound **35** was 4.6-fold (against HCT-116 cells), 1.3-fold (against BEL-7402 cells), and 2.6-fold (against HepG2 cells) more active than the positive control drug 5-fluorouracil. The compound was 18.6-fold (against HCT-116 cells), 6.3-fold (against BEL-7402 cells), and 11.2-fold (against HepG2 cells) more active than the lead compound isosteviol. Compound **35** also inhibited colony formation in HCT-116 cells in a concentration-dependent manner. Cell cycle analysis revealed that compound **35** inhibited cell growth via the induction of S phase arrest in HCT-116 cells. The possible mechanism of action may be correlated with downregulation of cyclin A and cyclin E1 expression and with the upregulation of cyclin B1 expression.

Oridonin (chemical formula: C_20_H_28_O_6_, molecular weight: 364.43, (1α,6β,7α,14 R)-7,20-Epoxy-1,6,7,14-tetrahydroxykaur-16-en-15-one) was initially isolated from various Isodon species that are commonly used as a home remedy herb in China and Japan. Derivatives of oridonin and triazole possess anti-tumour activity^99^. All oridonin derivatives containing different phenyl 1,2,3-triazoles exhibited stronger anti-proliferative activities against all three selected cancer cell lines than did oridonin and 5-Fu. Compound **36a** (Figure 9), with 4-methoxyphenyl 1,2,3-triazole, was the most potent compound in the series against the HCT116 cell line, with an IC_50_ value of 1.94 µM. This compound was approximately threefold more potent than oridonin against the tested cancer cell lines. Compound **36b** (Figure 9), with 4-methylphenyl 1,2,3-triazole, was the most potent compound in this series, with an IC_50_ value of 3.01 µM in MCF-7 cell lines. This compound was approximately sixfold more potent than oridonin against the tested cancer cell lines. Preliminary results suggested that the phenyl 1,2,3-triazole groups would improve the anti-proliferative activities of oridonin, and highlighting the importance of the linker.

Ferulic acid (chemical formula: C_10_H_10_O_4_, molecular weight: 194.18, 3–(4-Hydroxy-3-methoxyphenyl)-2-propenoic acid) is an abundant phenolic phytochemical found in plant cell walls. Aneja and et al.^100^ reported potent and selective ferulic acid-based small molecule inhibitors of carbonic anhydrase IX that possess significant inhibitory potential against various oncogenic parameters. The potency of compounds bearing –Cl, –NO_2_ and –COOH functionality was significantly enhanced in comparison to its natural precursor, ferulic acid with IC_50_ in the range of 0.024–3.78 µM. Fluorine substituent did not exhibit any significant effect on the activity against these carbonic anhydrase isoforms. However, the effect of electron-donating substituents on inhibition of carbonic anhydrase isoforms was found to be moderate only in the range of 1.96–6.67 µM. Interestingly, it was found that compound **37** (Figure 9) selectively inhibited carbonic anhydrase IX in the nanomolar range (IC_50_ = 24 nM). *In silico* analysis revealed the binding of compound **37** to the catalytically important amino acid residues of carbonic anhydrase IX. Further, cell-based studies indicated that compound **37** inhibits the activity of carbonic anhydrase IX, decreases epithelial to mesenchymal transition, induces apoptosis, and inhibits cell migration and colonisation potential in cancer cells. Taken together, these results emphasise the potential for use of compound **37** as a prospective pharmacological lead molecule in carbonic anhydrase IX-targeted anticancer therapeutics.

Derivatives of chalcones-triazole not only exert anti-breast cancer activity^60^^,^^61^, but also they exhibit activity against multiple types of cancer cells^101^^,^^102^. A new series of 1,2,3-triazole-chalcone hybrids was synthesised by Ashour and et al.^101^ The results suggest that the electronic and steric properties of the substituents play an important role in the binding affinity of chalcones to their cellular target(s). As a general statement, the presence of *meta* OCH_3_ group on the right side and *para* Cl atom on the left side of the hybrid was proved to be essential for general anticancer activity of the tested series. The para chloro compound **38** (Figure 9) that possesses a 3–4-dimethoxyphenyl chalcone moiety was the most potent derivative and inhibited the growth of RPMI-8226 and SR leukaemia cell lines by 99.73% and 94.95% at 10 µM, respectively. Furthermore, this compound inhibited the growth of M14 melanoma, K-562 leukaemia, and MCF7 breast cancer cell lines by more than 80% at the same test concentration. Compound **38** exhibited IC_50_ values of less than 1 µM against six types of tumour cells and possessed a high selectivity index that reached 104-fold in MCF7. Compound **38** possessed superior activity compared to that of methotrexate and gefitinib against the most sensitive leukaemia cell lines, and it exhibited higher or comparable activity against the other sensitive cell lines. Flow cytometry analysis of RPMI-8226 cells revealed that compound **38** caused cell cycle arrest at the G2/M phase and induced apoptosis in a dose-dependent manner. Mechanistic evaluation indicated that apoptosis induction triggered the mitochondrial apoptotic pathway by inducing ROS accumulation and increasing the Bax/Bcl-2 ratio and the activation of caspases 3, 7, and 9. The current study clearly identified the potential of compound **38** as a promising lead for the future development of active anticancer agents, and the results of this study may offer new insights for treating multiple myeloma based on the data generated using RPMI-8226 cells. A series of chalcone linked-1,2,3-triazoles was synthesised by Yadav and et al.^102^ All of the synthesised products were subjected to MTT cytotoxicity assays against a panel of four human cancer cell lines (MCF-7, MIA-Pa-Ca-2, A549, and HepG2) to assess their anticancer potential. Structure activity relationship results demonstrated the broad spectrum anticancer potential of compounds bearing methoxy substituents on ring A. In case of triazole derivative, the introduction of bromo or fluoro groups on benzene ring C led to decrease in anticancer activity compared to the parent compound **39** especially against MCF-7 and MIA-Pa-Ca-2 cell lines. Compound **39** exhibited more than 20-fold and 25-fold cytotoxicity towards the MCF-7 and MIA-Pa-Ca-2 cell lines as compared to normal cell lines fR2. All the synthesised triazoles were found to be less toxic towards the normal cells compared to the reference. Compound **39** (Figure 9) was found to be most active against all of the tested cancer cell lines, with IC_50_ values in the range of 4–11 µM. This compound also exhibited improved or comparable activity compared to that of the reference drug against all the tested cell lines. Cell cycle analysis revealed that compound **39** induces apoptosis and G2/S arrest in MIA-Pa-Ca-2 cells. Compound **39** triggers mitochondrial potential loss in pancreatic cancer MIA-Pa-Ca-2 cells Furthermore, compound **39** triggers caspase-3 and PARP-1 cleavage, and this cleavage increases in a dose-dependent manner.

Podophyllotoxin (chemical formula: C_22_H_22_O_8_, molecular weight: 414.41, (5 *R*,5a*R*,8a*R*,9*R*)-5,8,8a,9-Tetrahydro-9-hydroxy-5–(3,4,5-trimethoxyphenyl)furo[3′,4′:6,7]naphtho[2,3-*d*]-1,3-dioxol-6(5a*H*)-one) is a natural lignin and a natural product that is isolated from the roots of *Podophyllum hexandrum* growing in the wild. A series of new 4β-amidotriazole-linked podophyllotoxin derivatives was designed and synthesised by Reddy and et al.^103^ The majority of derivatives exhibited promising antiproliferative activity with IC_50_ values ranging from 1 to 10 µM in six human cancer cell lines that included cervical (HeLa), breast (MCF-7), prostate (DU-145), lung (A549), liver (HepG2), and colon (HT-29) cancer lines. The structure activity relationship indicates that the aryl triazolic-amide derivatives shown higher cytotoxicity in comparison to their corresponding benzyl triazolic amide derivatives, and the strong electron withdrawing groups like chloro, trifluoromethyl at position-4 in aryl triazolic-amide derivatives exhibited superior cytotoxicity than the standard. Among them, the congeners **40a**, **40b**, and **40c** (Figure 9) exhibited remarkable cytotoxicity, as indicated by IC_50_ values of <1 µM against the DU-145 cell lines. These compounds were found to be more active than etoposide. Moreover, compound **40b** exhibited remarkable cytotoxicity with IC_50_ values in the range of 0.70–4.11 µM, and this was the most promising compound in the series. For example, cervical (HeLa), breast (MCF-7), prostate (DU-145), lung (A549), liver (HepG2), and colon (HT-29) cancer cell lines were affected by compound **40b** with IC_50_ values of 0.78, 0.97, 0.70, 1.20, 0.78, and 4.11 µM, respectively. Similarly, compound **40a** exhibited IC_50_ values of 6.49, 1.10, 0.99, 1.61, 2.79, and 11.4 µM, while compound **40c** exhibited IC_50_ values of 1.21, 1.35, 0.89, 1.96, 1.21, and 4.40 µM against these human cancer cell lines (HeLa, MCF-7, DU-145, A549, HepG2, and HT-29), respectively. The IC_50_ of etoposide when used as a standard drug ranged from 1.62 to 2.84 µM. Topoisomerase-mediated DNA relaxation assay results revealed that the derivatives could efficiently inhibit the activity of topoisomerase-II. Flow cytometry analysis of DU-145 cells revealed that these compounds cause arrest at the G2/M phase of the cell cycle. Further apoptotic studies were also performed on these DU-145 cells, and the results indicated that this class of compounds could effectively induce apoptosis.

Eugenol (Chemical formula: C_10_H_12_O_2_, molecular weight: 164.2, 2-Methoxy-4-(2-propen-1-yl)phenol) is the primary active component of clove oil (75–90%)^104^. Taia et al.^105^ reported the synthesis of a new series of heterocyclic eugenol derivatives through the mixed condensation reaction of 1,3-dipolar azide and the oxide of p-chlorophenylnitrile on 4-allyl-2-methoxy-1-(prop-2-yn-1-yloxy) benzene. The monoadducts and bis-heterocyclic derivatives were active against the fibrosarcoma cell line and against lung and breast carcinoma cell lines. The hybrid compound **41** (Figure 9) showed the highest anticancer activity against all tumour cell lines, with IC_50_ values between 15.31 and 23.51 µM.

Hederagenin (chemical formula: C_30_H_48_O_4_, molecular weight: 472.7, (3β,4α)-3,23-Dihydroxyolean-12-en-28-oic acid) is a naturally occurring oleane-type pentacyclic triterpene. A series of novel aryl-1*H*-1,2,3-triazol-4-yl methylester and amide derivatives of the natural product hederagenin was synthesised by Rodríguez-Hernandez and et al.^106^ The cytotoxic activities of all compounds were screened against a panel of six human cancer cell lines using the well-established photometric sulforhodamine B assay. The majority of the compounds displayed higher levels of antitumor activity than did the parent hederagenin. These results indicate that the presence of a bulky group bonded to carbonyl-28 of the triterpene skeleton modulates their cytotoxic activity. 1,2,3-Triazolyl compounds having an ester group are generally more cytotoxic than compounds carrying an amide moiety. The ester derivatives **42a**, **42b**, and **42c** (Figure 9) that possess an *m*-bromo, *m*-chloro*, and m*-nitro substituent, respectively, were the most active compounds against all human cell lines tested, and they exhibited EC_50_ values ranging from between 3.2 and 4.0 µM for **42a**, between 3.1 µM and 4.0 µM for **42b**, and between 3.2 and 4.1 µM for **42c**. These results revealed that these compounds (**42a**, **42b**, and **42c**) are at least eightfold more active than is the parent hederagenin; however, they were not selective between malignant and non-malignant cells. The *ortho*-fluorobenzyl-1,2,3-triazolyl ester **42d** (Figure 9) was the most active compound against this cell line (EC_50_ = 1.6 µM**).** This compound **(42d**) also exhibited some selectivity in cytotoxicity (SI = 5.4; SI is defined as the quotient of EC_50_ values according to EC_50 [NIH 3T3]_/EC_50 [tumour cell line_]), discriminating between the cancer cell line HT29 and the non-malignant mouse fibroblast line NIH 3T3.

Oleanolic acid (chemical formula: C_30_H_48_O_3_, molecular weight: 456.71, (3β)-3-Hydroxyolean-12-en-28-oic acid) is a natural pentacyclic triterpenoid compound that is synthesised in many plants through the cyclisation of squalene^107^. A series of novel oleanolic acid-coupled 1,2,3-triazole derivatives was designed and synthesised by Wei and et al.^108^ The synthesised compounds were screened for anticancer activity against a panel of five human cancer cell lines using an MTT assay. A number of these compounds exhibited improved anticancer activity against the tested cancer cell lines compared to that of the positive controls 5-fluorouracil and oleanolic acid. Compounds with *p*-substitutions at an aromatic ring are more active than corresponding compounds without substitutions or substitutions at an *ortho-* or *meta-* position. Compounds with electron withdrawing groups at an aromatic ring are generally more active than compounds without substitutions or substitutions with an electron donating group at the identical position. Compound **43** (Figure 9) possessed strong inhibitory activity against A375-S2 and HT1080 cells, with IC_50_ values of 4.97 and 3.51 µM. A series of pharmacology experiments revealed that compound **43** significantly induced HT1080 cell apoptosis. This compound can serve as a promising lead candidate for further study.

### 2.2. Anti-inflammatory activity

**Figure 10.**
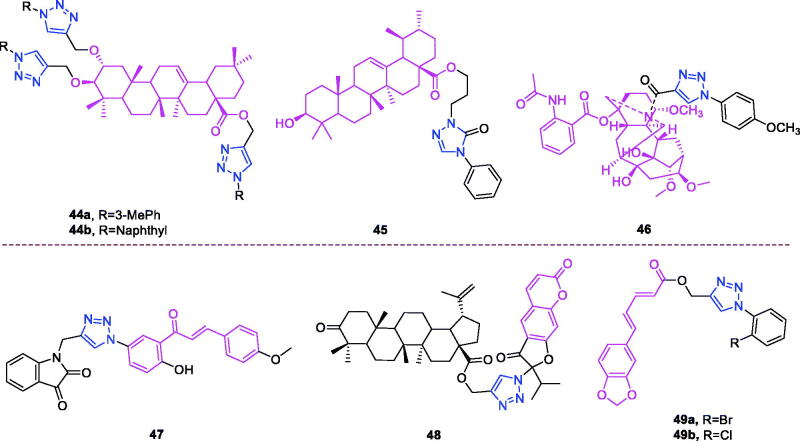
The chemical structures of anti-inflammatory compounds **44–49**.

Maslinic acid (chemical formula: C_30_H_48_O_4_, molecular weight: 472.7, (2α,3β)-2,3-Dihydroxyolean-12-en-28-oic acid) is a natural pentacyclic triterpene. In the European diet, olive oil and table olives represent a significant source of maslinic acid^109^. The introduction of triazole moieties into maslinic acid considerably improves its anti-inflammatory effect^110^. The tested compounds from the series of tri-1,4-disubstituted triazoles were found to be the most potent among the synthesised compounds (% IL-1β production = 23–47; 30–100 µM). This finding showed the importance of the number and may be also the position of the triazole moieties to improve the anti-inflammatory activity of maslinic acid. Compounds **44a** (*m*-Me) (Figure 10) and **44b** (Figure 10) possessing a naphthyl group on the triazole ring exhibited relatively high activities (% IL-1β production = 23 ± 3 and 34 ± 3, respectively; 30 µM) compared to those of the remaining analogs and of maslinic acid (% IL-1β production = 109 ± 3; 30 µM).

Ursolic acid (chemical formula: C_30_H_48_O_3_, molecular weight: 456.7, (3β)-3-Hydroxyurs-12-en-28-oic acid) is a well-known pentacyclic triterpene that is one of the major active components of many traditional Chinese medicines^111^. Most ursolic acid derivatives containing oxadiazole, triazolone, and piperazine moieties^112^ exhibited pronounced anti-inflammatory effects at 100 mg/kg. Compound **45** (Figure 10) showed the most potent inhibitory activity against ear inflammation of all the synthesised compounds (69.76%), and this activity was higher than that of UA (57.67%), ibuprofen (25.17%) and indomethacin (26.83%) at 100 µg/kg (i.p.) and was one- and twofold more potent than were the standard drugs. The cytotoxicity of the compounds was assessed using the MTT assay, and no compounds exhibited any appreciable cytotoxic activity (IC_50_ >100 lmol/L), which was in contrast to ursolic acid. These results indicate that incorporation of a triazolone moiety to ursolic acid can improve the anti-inflammatory activity, and the order of activity for the different carbon chain lengths was C3 > C4 > C2 > C5.

Lappaconitine (chemical formula: C_32_H_44_N_2_O_8_, molecular weight: 584.7, (1a,14a,16b)-20-Ethyl-1,14,16-trimethoxyaconitane-4,8,9-triol 4–(2-acetylamino)benzoate) is extracted from the roots of *Aconitum sinomontanum* Naka. A series of novel lappaconitine derivatives possessing various substituents at the 20-N position was designed and synthesised by Pang and et al.^113^ In the initial screening of lappaconitine derivatives against NO production, the target compounds exhibited excellent inhibitory ability relative to that of lappaconitine. In particular, compound **46** (Figure 10) exhibited the most potent inhibition, with an IC_50_ of 12.91 µmol/L. The elementary structure activity relationship of NO inhibitory activity indicated that replacement of the benzene ring with an electron donating group (4-(morpholine-4-yl) < 4–(4-phenylpiperazin-1-yl) < 4–(4-benzylpiperazin-1-yl) < 3,4,5-triOCH_3_ < 3,4-diOCH_3_ < 4-OCH_3_ < 4-(piperidin-1-yl)) could improve anti-inflammatory efficacy, and replacement of the benzene ring with an electron withdrawing group (4-CF_3_ < 4-F) could reduce anti-inflammatory efficacy. Furthermore, compound **46** exerts its anti-inflammatory activity by inhibiting NO, PGE2, and TNF-α generation *via* the suppression of NF-κB and MAPK signalling pathways. Notably, compound **46** could exert a significant therapeutic effect on LPS-induced acute lung injury (ALI) *in vivo*.

Chalcone-triazole derivatives not only possess anticancer activity^60^^,^^61^^,^^101^^,^^102^, but also they exhibit anti-inflammatory activity^114^. Boshra et al.^114^ synthesised new 2′-hydroxychalcone-triazole hybrids that possessed anti-inflammatory activity. The majority of the synthesised compounds exhibited anti-inflammatory activity that was equivalent to or even higher than that of celecoxib. The results showed that introducing electron donating group such as 3,4-dimethoxy or electron withdrawing group such as 4-Br to the phenyltriazole derivative improved the potency. Particularly, introducing lipophilic moiety as Cl or Br increases inhibitory potency as well as selectivity against COX-2. In order to investigate the effect of phenyltriazole moiety on activity, phenyl group was replaced by isatin to give compounds. Interestingly, some compounds of this series showed higher potency and selectivity for COX-2 than the corresponding phenyltriazole derivative. This improvement in potency of this series may be attributed to the synergistic effect of isatin moiety. An *in vitro* COX-1/COX-2 inhibition study revealed that among the synthesised compounds, compound **47** (Figure 10) exhibited the highest inhibitory activity against COX-2, with an IC_50_ value of 0.037 µM and a selectivity index of 359.46. Most of the compounds possessed significant *in vitro* 15-LOX inhibitory activity that was higher than that of zileuton. Therefore, compound **37** is a potent dual inhibitor of COX-2 and 15-LOX.

Coumarin-triazole derivatives not only possess anticancer activity^58^^,^^68^^,^^70^^,^^90–93^, but also they exhibit anti-inflammatory activity^115^. Lipeeva et al.^115^ synthesised conjugates of coumarin possessing lupan triterpenoids and 1,2,3-triazole and tested their anti-inflammatory activity. Among them, compound **48** (Figure 10), a conjugate of lupine triterpenoid with furocoumarin oreoselone, possessed marked anti-inflammatory activity. This compound significantly reduced paw edoema caused by the injection of histamine (edoema index: 24.5%) to a level that was comparable to that of the nonsteroidal anti-inflammatory drug indomethacin. Among the hybrids of betulonic acid with the coumarin peuruthenicin, compound containing an alkyl triazole linker with a short three-unit alkyl chain exhibited a weak anti-inflammatory effect. This compound statistically significantly reduced the histamine-induced edoema; its effect was two times weaker than the effect of conjugate (**48**). The elongation of the alkyl chain of the linker led to a loss of anti-inflammatory activity.

Piperine (chemical formula: C_17_H_19_NO_3_, molecular weight: 285.37, 5–(1,3-Benzodioxol-5-yl)-1–(1-piperidinyl)-2,4-pentadien-1-one) is a major alkaloid constituent of piper species, including *Piper nigrum Linn* and *Piper longum Linn*. This compound is commonly used in various traditional medicine systems. Nineteen novel piperine-based triazoles were synthesised using a click chemistry approach and were tested for *in vivo* anti-inflammatory activity^116^. The most active compounds were evaluated for *in vitro* TNF-α expression. Compounds **49a** and **49b** (Figure 10) were found to exert significant *in vivo* inhibition of inflammation at levels of 80.40% and 76.71%, respectively, after 5 h in comparison to that caused by piperine (54.72%) and the standard drug indomethacin (77.02%) without causing any damage to the stomach. Compounds **49a** and **49b** suppressed TNF-α levels by 73.73% and 70.64%, respectively, and reduced the protein expression of COX-2, NF-κB, and TNF-α to a greater degree than that caused by indomethacin. Moreover, compound **49a** was found to exert a significant analgesic activity of 54.09%, and this was comparable to that caused by indomethacin (57.43%). The structure activity relationship of the synthesised compounds has been analysed as follows. Presence of electron withdrawing groups on the aryl moiety decreased the in vivo anti-inflammatory activity as compared to the presence of electron donating groups. Better in vivo anti-inflammatory activity was observed for compounds having halogen at ortho position of the aromatic ring A as compared to halogen attached at para position. Increasing order of activity found was F < Cl < Br.

### 2.3. Antimicrobial activity

#### 2.3.1. Antibacterial activity

**Figure 11.**
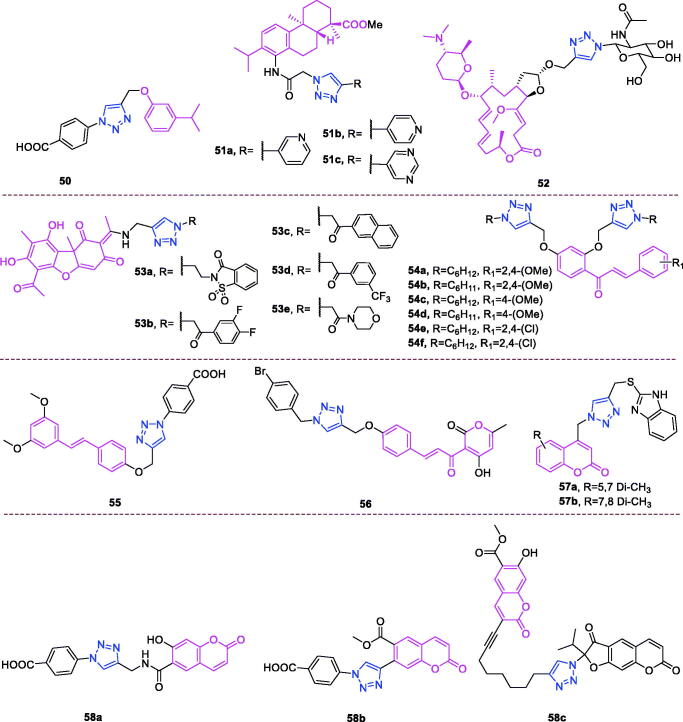
The chemical structures of antibacterial compounds **50–58**.

Carvacrol (chemical formula: C_10_H_14_O, molecular weight: 150.22, 2-Methyl-5–(1-methylethyl)phenol) is a monoterpene-phenol that is found in the essential oil of many aromatic plants of the Lamiaceae family, including thyme and oregano. Aneja et al.^117^ designed and synthesised 1,2,3-triazole analogs of natural bioactive precursors. These compounds exhibited moderate to potent antibacterial activity against both Gram-positive and Gramnegative bacteria. The analogues bearing electron-donating substituents such as *p*-methyl and *p*-methoxy exhibited better inhibition of all bacterial strains in comparison to halogensubstituted (*p*-fluoro and *p*-chloro substitution) analogues, where the activity was considerably lost against all strains. Among all of these triazole analogs, compound **50** (Figure 11) (derived from carvacrol) that possesses carboxylic acid functionality emerged as a potent antibacterial agent against *Streptococcus pneumoniae* (IC_50_: 62.53 µg/mL), *Enterococcus faecalis* (IC_50_: 36.66 µg/mL), and *Escherichia coli* (IC_50_: 15.28 µg/mL). Furthermore, compound **50** also demonstrated moderate efficacy against multidrug-resistant *Escherichia coli* strains. Compound **50** in combination with ciprofloxacin displayed a synergistic effect on the multidrug-resistant *Escherichia coli* MRA11 and MRC17 strains. Growth kinetics studies performed on *Streptococcus pneumoniae* and *Escherichia coli* treated with compound **50** revealed an extended lag phase. TEM analysis indicated that compound **50** caused significant cell wall damage and membrane disruption in bacterial cells (*Streptococcus pneumoniae* and *Escherichia coli*), ultimately leading to cell death. Moreover, this compound was also found to be a potent anti-biofilm agent against *Streptococcus pneumoniae* and *Escherichia coli* strains and exhibited non-cytotoxic effects on a human embryonic kidney (HEK293) cell line up to a concentration of 100 µg/mL. Additionally, this compound did not cause an alteration in haemocyte density, indicating the lack of an immune response, and it was also non-toxic to the larvae of *Galleria mellonella* up to a concentration of 2.5 mg/mL.

Dehydroabietane acid-triazole derivatives not only possess anticancer activity^59^^,^^69^, but also they exhibit antibacterial activity^118^. Hou et al.^118^ obtained a diverse natural product-like synthetic abietane diterpenoid library containing 86 compounds. The structure activity relationships showed that the introduction of 1,2,3-triazole ring at the C-14 position of dehydroabietic acid could retain the potent antibacterial activities. Generally, the introduction of electron-poor aromatic ring system on the C-4 position of the 1,2,3-triazole moiety would significantly increase the antibacterial activities. In particular, compounds **51a**, **51b** and **51c** (Figure 11) showed promising low MICs against the tested bacteria. While, with the saturated alkyl-, ester- and trimethylsilyl- substituted groups on the C-4 position of the 1,2,3-triazole moiety there were no significant structure activity relationships. Among them, compound **51c** exhibited the most potent activities against both gram-positive bacteria (*Bacillus subtilis* and *Staphylococcus aureus*) and gram-negative bacteria (*Escherichia coli* and *Pseudomonas fluorescens*) with comparable MICs (1.6 µg/mL to 3.1 µg/mL) to those observed in response to the positive control norfloxacin. More importantly, compound **51c** exhibited very low cytotoxicity (CC_50_: 31.7 µg/mL and 41.8 µg/mL) against normal human foreskin fibroblast (HFF) and liver (HL-7702) cells.

Spiramycin (chemical formula: C_43_H_74_N_2_O_14_, molecular weight: 843.05, Spiramycin) is a natural antibiotic produced by *Streptomyces ambofaciens* in the form of a mixture of three compounds known as spiramycins I–III^119^^,^^120^. Huisgen cyclo-addition allowed for the acquisition of novel triazole-bridged antibiotics possessing the reconstructed C(5) arm of spiramycin^121^. Further comparison of the physicochemical parameters of these compounds possessing a relatively hydrophilic C(5) arm revealed good solubility for all of these derivatives. Antibacterial activity studies demonstrated that the most active among novel triazole conjugates is that containing the terminal N-acetylsaccharide moiety (compound **52**). Compound **52** (Figure 11) possesses a lipophilicity that is similar to that of spiramycin, and this contributes to its generally comparable activity to that of spiramycin (is 2–4 less active than spiramycin [µM]). All of these results clearly show that 16-membered macrolide derivatives, even those not containing an aldehyde group but having additionally functionalised saccharide, can be active against Gram-positive bacteria at a level comparable to that of spiramycin.

Usnic acid (chemical formula: C_18_H_16_O_7_, molecular weight: 344.32, (9b*R*)-2,6-Diacetyl-7,9-dihydroxy-8,9b-dimethyl-1,3(2*H*,9b*H*)-dibenzofurandione) is a secondary metabolite derived from lichen sources such as *Usnia, Ramalina*, and *Cladonia*^122^^,^^123^. Usnic acid enaminone-coupled 1,2,3-triazole can be used as an antibacterial and antitubercular agent^124^. Among the synthesised derivatives, the most active analog (**53a**) (Figure 11) inhibited *Mycobacterium tuberculosis* (*Mtb*) at an MIC value of 2.5 µM, and this was slightly better than that of the standard reference isoniazid (2.9 µM). Compounds **53b** (3,4-difluorobenzoyl) (Figure 11) and **53c** (2-acylnaphthyl) (Figure 10) proved to be effective and exhibited MIC values of 5.4 and 5.3 µM, respectively. In contrast, the uniqueness of the synthesised triazoles was observed for *Bacillus subtilis*, where compound **53d** [3-(trifluoromethyl) phenacyl] (Figure 11) exhibited the greatest antibacterial efficacy (zone of inhibition of 15 mm) while also possessing moderate anti-tuberculosis activity. In addition to its anti-tuberculosis activity, compound **53e** (N-acylmorpholinyl) (Figure 11) also exhibited a 13 mm diameter inhibition zone against *Bacillus subtilis*. The active anti-tuberculosis compounds **53b** (3,4-difluorobenzoyl) and **53c** (2-acylnaphthalene) exert bacteriostatic effects on *Bacillus subtilis*, with inhibition zones of 12 and 11 mm, respectively. A structure–activity relationship assessment suggests that the presence of the fluorine atoms in **53b** might be a contributing factor for the increased potency of the molecule. Naphthalene substituted triazole (**53c**) displayed activity similar to that of **53b**, indicating that the *meta*- and *para*-positions should be occupied for antitubercular activity. The presence of a *p*-halogen is important and favourable, but the presence of an *m*-halogen might be a deciding factor for increased potency. The presence of fluorine is more favourable than chlorine. The presence of an electron-donating methoxy group is unfavourable for antitubercular activity. The present study shows that a versatile bridging unit like triazole, when coupled to usnic acid, might be beneficial in imparting an antitubercular property.

Chalcone-triazole derivatives not only possess anticancer activity^60^^,^^61^^,^^101^^,^^102^ and anti-inflammatory activity^114^, but also they exhibit antibacterial activity^125–127^. The newly synthesised bis-1,2,3-triazole based chalcones were tested for their antibacterial activity^125^. The compounds demonstrated significant inhibition of tested gram positive and gram negative strains compared to the standard drug Gentamicin sulphate. The compounds **54a**, **54b**, **54c**, **54d**, **54e**, and **54f** (Figure 11) demonstrated high antibacterial activity at concentrations of 75 and 100 µg/mL. The accumulated data indicated that presence of the strong electron donating group (OMe) at ortho and para positions of phenyl ring of chalcone could enhance the antibacterial activity of the other products. In contrast, triazolyl-pterostilbene derivatives exhibited potent antibacterial activity, particularly against methicillin-resistant *Staphylococcus aureus* (MRSA)^126^. Among these compounds, compound **55** (Figure 11) exhibited the most potent anti-MRSA activity with a minimum inhibitory concentration (MIC) value of 1.2–2.4. µg/mL and a minimum bactericidal concentration (MBC) value of 19.5–39 µg/mL. Anti-MRSA mechanism studies indicated that active compounds may inhibit MRSA by acting on DNA polymerase instead of the bacterial cell wall and cell membrane. The structure activity relationship indicated that the carboxylic acid must be the essential moiety in the antibacterial activity. And the spacer between triazole and carboxylic acid plays an important role in the inhibition of MRSA. Phenyl groups with the characteristics of planarity and resonance would be the most favourable spacer. A series of new dehydroacetic acid chalcone-1,2,3-triazole hybrids was designed, synthesised, and characterised for use as potential antimicrobial agents by Lal et al.^127^. Preliminary studies revealed that a number of the compounds exhibited bioactivities that were comparable to or even superior to those of the reference drugs. All of the triazole derivatives containing a substituted benzene ring displayed improved activity compared to that of dehydroacetic acid and dehydroacetic acid-chalcone alkyne, thereby highlighting the role of the 1,2,3-triazole moiety on the antimicrobial potential of the target compounds. Compounds containing bromo and methoxy groups on benzene ring exhibited better activity against most of the studied microorganisms. Compound **56** (Figure 11) was found to be the most active against *Escherichia coli*, with an MIC value of 0.0030 µM/mL. Additionally, molecular modelling studies indicated that compound **56** binds effectively to the active sites of DNA gyrase via hydrogen bonding, hydrophobic interactions, and electrostatic interactions.

Coumarin-triazole derivatives not only possess anticancer activity^58^^,^^68^^,^^70^^,^^90–93^ and anti-inflammatory activity^115^, but also they exhibit antibacterial activity^128^^,^^129^. A new class of triazole-linked coumarinyl 2-mercaptobenzimidazole hybrids was synthesised and screened for their anti-tuberculosis potential against MTB H_37_Rv by Anand et al. ^128^. All conjugates exhibited promising anti-mycobacterial activity against MTB H_37_Rv with MIC values ranging from 3.8 to 59.6 mM. In particular, the most active bis-substituted hybrids (**57a** and **57b**) (Figure 11) (MIC: 3.8 µM) were found to be 2.4- to 6.6-fold more potent than were the references Pyrazinamide, streptomycin, and ciprofloxacin (MIC: 25.3, 10.7, and 9.4 µM, respectively). Thus, hybrids **57a** and **57b** may become useful as new potential anti-tuberculosis agents in the future. Lipeeva et al.^129^ synthesised a new series of coumarinotriazole compounds. The synthesised coumarino-triazole-type derivatives were screened for their *in vitro* antimicrobial activity. Compounds **58a** and **58b** (Figure 11) possessing a 4-(carboxyphenyl) triazolyl substituent at the 6 or 7 position of the coumarin ring exhibited excellent antibacterial activity against *Staphylococcus aureus* strains, with MIC values of 0.16–3.75 µg/mL and 0.21–6.28 µg/mL, respectively. The coumarin-2,3-dihydrofurocoumarin hybrid compound **58c** (Figure 11) was found to be selective against *Bacillus subtilis* and *Escherichia coli*, with MIC values of 0.02–0.15 µg/mL. A molecular docking study was performed for the most active compounds against the MurB protein, and the molecular docking results were consistent with the *in vitro* antibacterial activity findings.

#### 2.3.2. Antifungal activity

**Figure 12.**
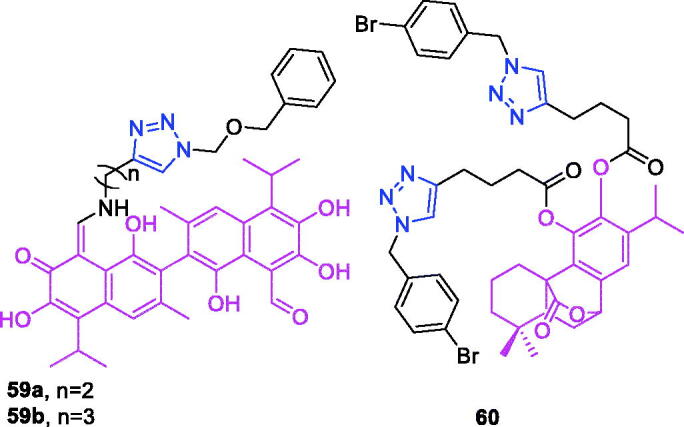
The chemical structures of antifungal compounds **59–60**.

Gossypol (chemical formula: C_30_H_30_O_8_, molecular weight: 518.55, 1,1′,6,6′,7,7′-Hexahydroxy-3,3′-dimethyl-5,5′-bis(1-methylethyl)[2,2′-binaphthalene]-8,8′-dicarboxaldehyde) is a yellow pigment that is present in various parts of cotton plants where it acts as a plant defense system against pathogenic fungi and insects^130^. This compound is a natural bisesquiterpene. Pyta et al.^131^ designed and synthesised novel gossypol triazole conjugates that were functionalised with aliphatic chains and benzyloxy groups. Biological evaluation of the new gossypol-triazole conjugates revealed that the potency of the **59a** and **59b** derivatives (Figure 12) possessing triazole-benzyloxy moieties was comparable to that of miconazole against *Fusarium oxysporum* (MICs = 16 µg/mL). The results of HPLC evaluation of ergosterol content in different fungal strains upon treatment with gossypol and its derivatives indicate that the mechanism of antifungal activity of gossypol and its triazole-containing derivatives may be involve the inhibition of ergosterol biosynthesis, as this process is crucial for controlling the permeability and fluidity of fungal plasma membranes in a manner similar to that of cholesterol in animals.

Carnosol (chemical formula: C_20_H_26_O_4_, molecular weight: 518.55, (4a*R*,9*S*,10a*S*)-1,3,4,9,10,10a-Hexahydro-5,6-dihydroxy-1,1-dimethyl-7–(1-methylethyl)-2*H*-9,4a-(epoxymethano)phenanthren-12-one) is one of the major phenolic diterpenes derived from the leaves of *Rosmarinus officinalis* L. Pertino et al.^132^ used click chemistry to synthesise a series of twenty-four novel abietane diterpene derivatives with good to reasonable yields. The antifungal activity of the compounds was determined as the percentage of inhibition of *Candida albicans* ATCC 10231 and *Cryptococcus neoformans* ATCC 32264 in the range of 250–3.9 µg/mL. From these data, the MIC_100_ and MIC_50_ were determined for all of the synthesised compounds. The most active carnosol derivative was the *p*-bromobenzyl derivative **60**, which reduced the growth of *Cryptococcus neoformans* by about 91.3% at 250 µg/mL while compound, with a *p*-nitrobenzyl unit decreased fungal growth by about 71% at the same concentration. The results indicate some selectivity for the different fungi and that the placement of the lactone (either C-20, C-11 or C-20, C-7) is important for the effect.

#### 2.3.3. Miscellaneous

**Figure 13.**
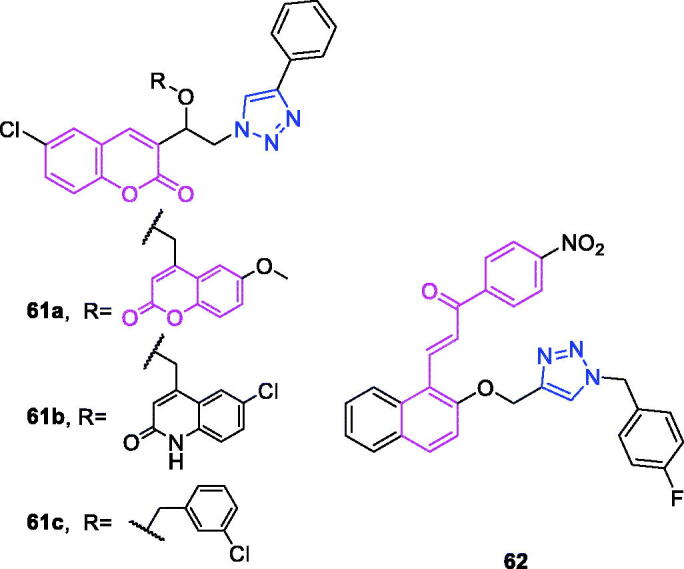
The chemical structures of anti-multiple timicrobial compounds **61–62**.

Coumarin-triazole derivatives not only possess anticancer activity^58^^,^^68^^,^^70^^,^^90–93^, anti-inflammatory activity^115^, and antibacterial activity^128^^,^^129^, but also they exhibit antifungal activity^133^. A series of new coumarin-, quinolinone-, and benzyl-linked 1,2,3-triazole derivatives were synthesised and screened for their antibacterial and antifungal activities by Savanur and et al.^133^ Most of the compounds exhibited good activity in against the Gram-positive bacteria *Staphylococcus aureus* and *Bacillus subtilis* and the Gram-negative bacteria *Pseudomonas aeruginosa*. Almost all of the compounds exhibited very good antifungal activity. Most of them were highly active against *Candida albicans*, *Candida tropicalis*, *Candida utilis*, and *Candida krusei* and exhibited moderate activity against *A. fumigatus*, *A. niger*, *R. oryzae*, and *R. bataticola*. Compounds possessing chloro and methoxy substitution in coumarin (**61a**) (Figure 13) were found to be very active against *Staphylococcus aureus*, *Pseudomonas aeruginosa*, *Candida albicans*, *Candida utilis*, and *Candida krusei*, with an MIC of 1 µg/mL that was similar to that of the standard. This compound also exhibited good activity against *Bacillus subtilis*, *Bacillus cereus*, and *Bacillus bronchiseptica*, with MIC values of 8 µg/mL and 16 µg/mL, respectively. The same compounds also showed good anti-fungal activity in other highly resistant fungi. Compounds possessing chloro substitution at C-6 in coumarin and 1-aza coumarin (**61b**) (Figure 13) were highly effective against *Pseudomonas aeruginosa* and *Candida tropicalis*, with an MIC of 1 µg/mL. These compounds also exhibited very good activity against *Staphylococcus aureus*, with an MIC of 4 µg/mL. Furthermore, chloro-substituted triazoles possessing a benzyl group (**61c**) (Figure 13) exhibited excellent activity against *Staphylococcus aureus* and *Candida albicans*, with an MIC of 1 µg/mL that was similar to that of the standard. Further structural activity relationship studies indicated that the compounds bearing methoxy, chloro and methyl substitution in coumarin, and quinolinone, enhances the anti-microbial activity and more selectively bearing bis-chloro substitution compounds showed excellent anti-fungal activity. It can be inferred that electron withdrawing or donating character of the substituents does not seem to be a major factor in increasing or decreasing anti-microbial activity. Thus, these compounds represent new platform that can be further optimised to seek novel anti-fungal agents with structures significantly different from those of existing anti-microbials.

Chalcone-triazole derivatives not only possess anticancer activity^60^^,^^61^^,^^101^^,^^102^, anti-inflammatory activity^114^, and antibacterial activity^125–127^, but also they exhibit antifungal activity^134^. Yadav et al.^134^ synthesised new fluorinated-chalcone-1,2,3-triazoles possessing potential antimicrobial activity. Preliminary *in vitro* antibacterial screening indicated that the majority of the synthesised compounds exhibited good activity. Compound **62** (Figure 13) possessing a 4-nitro group was found to be more active than was the standard, with an MIC value of 0.0032 mmol/mL against *Escherichia coli* and *Staphylococcus epidermidis*. For *Pseudomonas aeruginosa*, compound **62** was found to be more active than all of the other tested compounds, with an MIC value of 0.0063 µmol/mL. The majority of the synthesised hybrids also exhibited good antifungal activity. Compound **62** possessed higher potency compared to that of fluconazole (MIC = 0.0102 µmol/mL) against *A. niger* and *C. albicans*, with an MIC value of 0.0032 µmol/mL. Structure activity relationship：i) Most of the fluorinated triazoles exhibited good results than the non-fluorinated compound. ii) The synthesised triazole analogues with a substituted benzene showed better activity than naphthaldehyde-chalcone alkynes, exhibiting the significance of 1,2,3-triazole. These outcomes revealed the additive effect of biological activity when two pharmacophoric moieties, i.e. chalcone and 1,2,3-triazole are conjugated. iii) Compounds with electron withdrawing substituents on benzene displayed superior activity than having electron releasing groups. iv) It was observed that compounds with nitro and methoxy substituents on benzene ring demonstrated better activity against majority of the microorganisms under test. v) Molecule **62** containing *p*-nitro group exhibited good antifungal activity and was more effective than Fluconazole. vi) Activity results also revealed that most of the triazole hybrids exhibited superior antifungal potency compared to antibacterial activity.

### 2.4. Antiparasitic activity

#### 2.4.1. Anti-leishmanial activity

**Figure 14.**
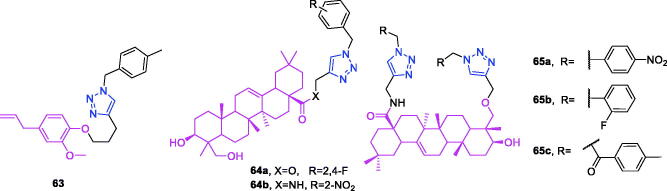
The chemical structures of anti-leishmanial compounds **63–65**.

Eugenol-triazole derivatives not only possess anti-cancer activity^105^, but also they exhibit anti-leishmanial activity^135^. Eugenol derivatives exert leishmanicidal activities with varying degrees of effectiveness^135^. The most active compound, 4–(3-(4-allyl-2-methoxyphenoxy)propyl)-1–(4-methylbenzyl)-1*H*-1,2,3-triazole (**63**) (Figure 14) (IC_50_ = 7.4 ± 0.8 µmol/L), also targeted *Leishmania* parasites inside peritoneal macrophages (IC_50_ = 1.6 µmol/L) without interfering with cell viability. The cytotoxicity of compound **63** against macrophage cells was indicated by an IC50 of 211.9 µmol/L and a selective index of 132.5. Under similar conditions, compound **52** was more effective than were glucantime and pentamidine, two drugs currently used in the clinic. Theoretical calculations indicated that this compound also exerts the majority of its physicochemical and pharmacokinetic activities within the ranges expected for orally available drugs. It is believed that eugenol bearing 1,2,3-triazole functionalities may represent a scaffold to be explored towards the development of new agents to treat leishmaniasis.

Hederagenin-triazole derivatives not only possess anti-cancer activity^106^, but also they exhibit anti-leishmanial activity^136^^,^^137^. A series of hederagenin derivatives synthesised by Rodríguez-Hernández and et al exert highly potent anti-leishmanial effects^136^. Some derivatives possessed activity at the micromolar level and exhibited low toxicity against BGM and HepG2 cells. Moreover, the ability of the hederagenin derivatives **64a** (11 µM) and **64b** (2 µM) (Figure 13) to prevent the proliferation of intracellular amastigote forms of *Leishmania infantum* and their higher selectivity index and low toxicity compared to commercial positive control drugs (potassium antimonyl tartrate trihydrate) (IC_50_ = 80 µM, SI = 0.1) make these compounds promising candidates for the treatment of leishmaniasis. Although a considerable number of hederagenin derivatives, carrying or not a triazol unit, bonded with an aromatic system with several electron donating and withdrawing groups at different positions, have been prepared, no structural-activity relation could be clearly established. Rodríguez-Hernández et al.^137^ also found that derivatives **65a**, **65b**, and **65c** (Figure 13) were highly effective in preventing the proliferation of intracellular amastigote forms of *Leishmania* infantum (IC_50_ = 25.9, 5.6, and 7.4 µM, respectively). All of these compounds exhibited a higher selectivity index and a low toxicity against two strains of kidney (BGM) and liver (HepG2) cells. Compound **65b** possessed a higher selectivity (1780 times) in comparison to that of the commercial antimony drug and is approximately 8 times more selective than is the most active compound previously reported as a hederagenin derivative. Additionally, hederagenin and certain derivatives (**65a**, **65b**, and **65c**) exhibited interactions with the binding site of the enzyme CYP51_Li_. In general the most active compounds bear a substituent at *para* or *ortho* position. None of the compounds with a *meta*-substituent had a significant activity. On the other hand, revealed no clear influence of the extra C = O moiety attached to the phenyl ring on the activity.

#### 2.4.2. Antimalarial activity

**Figure 15.**
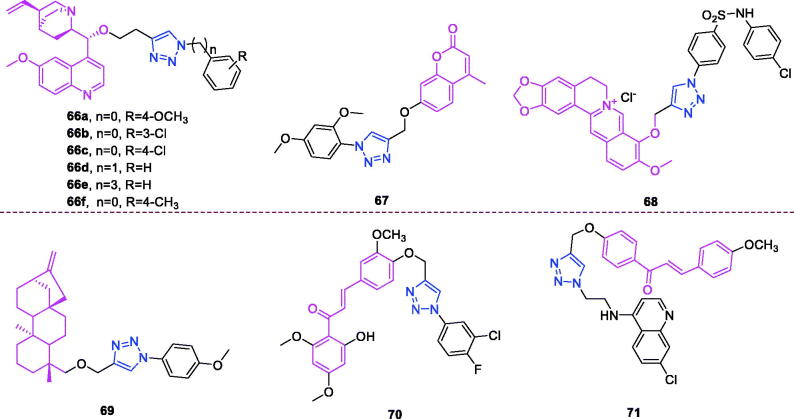
The chemical structures of antimalarial compounds **66–71**.

Quinine (chemical formula: C_20_H_24_N_2_O_2_, molecular weight: 324.42, (8α,9 *R*)-6′-Methoxycinchonan-9-ol) is the most abundant Cinchona alkaloid and was the only known antimalarial drug for over 300 years^138^. Faidallah et al.^139^ synthesised a variety of 1,2,3-triazole-quinine conjugates. The synthesised compounds were bio-assayed against the blood stage of *Plasmodium falciparum* strain 3D7 according to the *in vitro* standard procedure. All the amino acid and dipeptide analogs were found to be less potent than quinine. Some of the aryl and heteroaryl analogs showed more promising submicromolar potency. Upon inserting a second methylene between quinine and triazole moieties, the potency dramatically improved. A number of the synthesised analogs (IC_50_ = 43, 37, 41, 40, 30, and 27 nM for compounds **66a**, **66b**, **66c**, **66d**, **66e**, and **66f**, [Figure 15], respectively) exhibited antimalarial properties with higher potency than that of the starting precursor quinine (standard reference used). This may be attributed to the lipophobic/hydrophilic properties of long alkyl chain containing compounds.

Coumarin-triazole derivatives not only possess anticancer activity^58^^,^^68^^,^^70^^,^^90–93^, anti-inflammatory activity^115^, antibacterial activity^128^^,^^129^, and antifungal activity^133^, but also they exhibit antimalarial activity^140^. Coumarin-triazole analogs possess antiplasmodial efficacy *in vitro*^140^. The results show that most of the new compounds of coumarin-triazole series exhibited very promising activity ranges, depending on the substitution on position-1 of triazole ring, like alkyl chain having polar group and phenyl ring having different functional group. The presence of electron releasing group and electron withdrawing group, their position (ortho, meta and para) in phenyl ring and their steric hindrance, all the factors might affect the activity, but varying behaviour of compounds with similar nature of functional groups is still elusive. Among these compounds, compound **67** (7-[1–(2, 4-dimethoxy-phenyl)-1*H*-[1–3] triazol-4-ylmethoxy]-4-methyl-chromen-2-one) (Figure 15) having 2, 4-dimethoxy phenyl ring was the most active, with an IC_50_ value of 0.763 ± 0.0124 µg/mL, presence of two electron releasing methoxy group at ortho and para position of phenyl ring enhanced the antimalarial activity quite significantly. Convincing results were obtained by analysing their inhibitory effect on the supercoiling activity of the enzyme gyrase through assays that employed the use of *Escherichia coli* DNA gyrase and relaxed plasmid DNA. Thus, these compounds can be used as potential agents to synthesise new antimalarial drugs that target the DNA gyrase enzyme.

Berberine-triazole derivatives not only possess anticancer activity^87^, but also they exhibit antimalarial activity.^41^ Novel sulphonamide-based berberine-[1–3]-triazole hybrids were successfully synthesised by Batra and et al.^41^ The majority of the synthesised compounds possessed significant antimalarial activity, with IC_50_ values in the range of 0.1–20 µg/mL. These compounds were also found to be non-cytotoxic under the tested conditions. The majority of synthesised sulphonamide based berberine-triazole hybrids are proved to be significantly active against *P. falciparum*. Among the halogen-substituted derivatives, compound **68** (Figure 15) containing *a p*-chlorophenylamino substituent was determined to be the most active molecule, with an IC_50_ of 0.1 µg/mL.

Kaurenoic acid (chemical formula: C_20_H_30_O_2_, molecular weight: 302.455, 4α)-Kaur-16-en-18-oic acid) is a widely-occurring diterpenoid^141^. Santos et al.^142^ reported the synthesis of hybrid kauranoid molecules of type 1,2,3-triazole-1,4 disubstituted with the aim of improving the antimalarial activity of kaurenoic and xylopic acids. A total of nine novel hybrid kauranoid-1,2,3-triazole derivatives were obtained via the CuAAC reaction, although the products were much less active than were the starting diterpene acids. Derivative **69** (Figure 15) possessed the highest selective index (SI = 22.2). Chloroquine exhibited a CC_50_ value of 543.6 ± 71.4 µM, and the SI was 1308. Derivative **69** possessed CC_50_ values (> 2102.3 µM) that were greater than those of chloroquine, Specifically, the cytotoxicity of these substances as tested in HepG2 cells was significantly lower than that of chloroquine. However, the SI value of chloroquine was higher than the values found for derivative **69** due to its higher activity. Consequently, its IC_50_ is much lower. In this case, the presence of nitrogens, and possibly of protonated forms, would certainly influence in receptors interaction and, consequently, in the antiplasmodial effect.

Chalcone-triazole derivatives not only possess anticancer activity^60^^,^^61^^,^^101^^,^^102^, anti-inflammatory activity^114^, antibacterial activity^125–127^, and antibacterial activity^134^, but also they exhibit antimalarial activity^143^^,^^144^. Kant et al.^143^ attempted to synthesise a new class of triazole chalcone derivatives possessing antiplasmodial activity through the use of copper-catalyzed click chemistry. A number of these compounds exhibited moderate activities. The most potent compound was compound **70** (Figure 15) that possessed a 3-chloro-4-fluoro substituted benzene ring, ass this compound exhibited an IC_50_ of 2.74 µg/mL *in vitro* against the erythrocytic stages of *Plasmodium falciparum* (3D7 strain). These compounds were also evaluated for cytotoxicity *in vitro* against the Huh-7 cell line, and they exhibited no cytotoxic activity and CC_50_ values that were higher than 100 µg/mL. This is a preliminary result and to reach more appropriate conclusion 2nd and 3rd generation compounds should be synthesised in order to establish meaningful structure activity relationship. Chalcone-quinoline hybrids possessing aminoethylene and aminopropylene linkers between quinoline and 1,2,3-triazole moieties also showed potential *in vitro* antiplasmodial activities against the CQR W2 strain of *Plasmodium falciparum*^144^. The structure activity relationship results demonstrated that the shorter aminoethylene linker between quinoline and 1,2,3-triazole motifs was more potent than the longer aminopropylene linker between the quinoline and 1,2,3-triazole motifs. The most active hybrid (**71**) (Figure 15) with an IC_50_ of 114.1 nM against the CQR W2 strain of *P. falciparum* was not inferior to chloroquine (IC_50_: 150 nM), and it (CC_50_: 35.6 µM) also showed low cytotoxicity towards HeLa cells and exhibited a good selectivity index (SI: 311).

#### 2.4.3. Anti-Toxoplasma gondii activity

**Figure 16.**
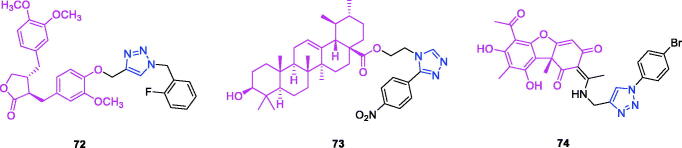
The chemical structures of anti-*Toxoplasma gondii* compounds **72–74**.

Arctigenin (chemical formula: C_21_H_24_O_6_, molecular weight: 372.41, (3 *R*,4*R*)-4-[(3,4-Dimethoxyphenyl)methyl]dihydro-3-[(4-hydroxy-3-methoxyphenyl)methyl]-2(3*H*)-furanone) is derived from the dried ripe fruit of *Arctium lappa*. Four new series of arctigenin derivatives were designed, synthesised, and evaluated for their anti-*Toxoplasma gondii* activity *in vitro* and *in vivo* by Zhang and et al.^145^ For the different substituted phenyl or benzyl 1,2,3-triazole compounds, the introduction of electron-withdrawing groups, such as halogens, at the *ortho* and *meta* positions of the benzene ring enhanced the ability of Hela cells to resist *Toxoplasma gondii*. However, the introduction of a halogen at the para position of the benzene ring was detrimental to the increase of anti- *Toxoplasma gondii* activity. On the other hand, the anti-*Toxoplasma gondii* ability was enhanced after the introduction of electron-donating groups to the para position of the benzene ring. Among the synthesised compounds, 4–(3,4-dimethoxybenzyl)-3–(4-((1–(2-fluorobenzyl)-1*H*-1,2,3-triazol-4-yl)methoxy)-3-methoxybenzyl)dihydrofuran-2(3*H*)-one (**72**) (Figure 16) exhibited the most potent anti- *Toxoplasma gondii* activity and a low cytotoxicity (IC_50_ in *Toxoplasma gondii*: 17.1 µM; IC_50_ in HeLa cells:≥ 600.0 µM; Selectivity: 35.09), thus yielding improved results compared to those from the lead compound arctigenin (IC_50_ in *Toxoplasma gondii*: 586.4 µM; IC_50_ in HeLa cells: 572.7 µM; Selectivity: 0.98) and the clinically applied positive-control drug spiramycin (IC_50_ in *Toxoplasma gondii*: 262.2 µM; IC_50_ in HeLa cells: 189.0 µM; Selectivity: 0.72). Compound **72** not only significantly reduced the number of tachyzoites in the peritoneal cavity of mice, but also it resulted in their partial malformation (*p* < 0.05) *in vivo*. Additionally, the results of a docking study of compound **72** into the *Toxoplasma gondii* calcium-dependent protein kinase 1 (TgCDPK1) receptor protein-binding site revealed that its mode of action was possibly as a TgCDPK1 inhibitor.

Ursolic acid-triazole derivatives not only possess anti-inflammatory activity^112^, but also they exhibit anti*-Toxoplasma gondii* activity^146^. Ursolic acid derivatives displayed some anti-*Toxoplasma gondii* activity and exhibited less cytotoxicity than ursolic acid *in vitro*^146^. The compounds with 1,2,4-phenyltriazole showed considerably higher anti- *Toxoplasma gondii* activity. It seems that the anti-*Toxoplasma gondii* ability was enhanced after the introduction of strong electronwithdrawing group (–F, –NO_2_) to the para position of the benzene ring. Compound **73** (Figure 16) exhibited the most potent anti-*Toxoplasma gondii* activity *in vivo* (Selectivity: 1.77) and was superior to ursolic acid (selectivity: 0.62) and the positive control spiramycin (Selectivity: 0.72). Additionally, determination of biochemical parameters, including the liver and spleen indexes, indicated that compound **73** effectively reduced hepatotoxicity and significantly enhanced anti-oxidative effects compared to those values in response to ursolic acid. Furthermore, a molecular docking study indicated that compound **73** possesses a strong binding affinity for *Toxoplasma gondii* calcium-dependent protein kinase 1.

Usnic acid-triazole derivatives not only possess antibacterial activity^124^, but also they exhibit anti*-Toxoplasma gondii* activity^147^. Six series of (+)-usnic acid derivatives were synthesised by Guo et al. ^147^. *In vitro*, the majority of the derivatives tested in this study exhibited more anti- *Toxoplasma gondii* activity than did the parent compound (+)-usnic acid and the positive control drugs. Compounds introduced different substituted aminotriazoles into the lead (+)-usnic acid, and the sequence of the selectivity index was as follows: p-Br > p-CF3 > p-F > o-Cl > o-OCH3> p-OCH3 > p-H > p-Cl. To some extent, this suggests that for the anti-*Toxoplasma* gondii activity it may be beneficial to introduce strong electron withdrawing groups in the para position of the benzene ring. The selectivity index of the triazole-linked (+)-usnic acid derivatives was 0.41–1.34. Among them, derivative **74** (Figure 16) exhibited the best anti-*Toxoplasma gondii* activity (selectivity: 1.34), and this activity was greater than that of the positive control drugs sulfadiazine (selectivity: 1.15), pyrimethamine (selectivity: 0.89), and spiramycin (selectivity: 0.72) and the lead compound (+)-usnic acid (selectivity: 0.96).

#### 2.4.4. Anti- Trypanosoma cruzi activity

**Figure 17.**
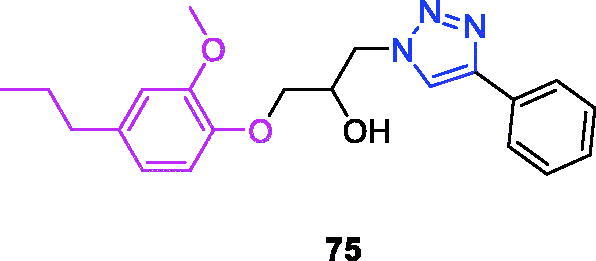
The chemical structure of anti-*Trypanosoma cruzi* compound **75**.

Eugenol-triazole derivatives not only possess anticancer activity^105^ and anti-leishmanial activity^135^, but also they exhibit anti-Trypanosoma *cruzi* activity^148^. De Souza et al.^148^ reported the synthesis of 1,2,3-triazoles obtained from eugenol and di-hydroeugenol and their *in vitro* and *in vivo* trypanocidal activity. Compound **75** exhibited the highest activity against the epimastigote forms of *Trypanosoma cruzi* (Y strain) (IC_50_ = 42.8 µM) and were weakly toxic to cardiomyoblast cells (H9c2 cells), similarly to that presented by the control drug benznidazole. Although this derivative had a lower SI than benznidazole, it can be considered an innovative structural core for optimisation and design of new tripanocidal agents. Moreover, the triazole **75** was twice as active as the corresponding eugenol derivative (IC_50_ = 88.4 µM) pointing the importance of the *n*‐propyl side chain for this activity. It is possible to note among these 1,2,3‐triazoles that the phenyl group was the best substituent at the triazole core, because derivatives with hydroxymethyl, acetyl or cyclohexyl groups showed lower or no trypanocidal activity. Compounds **75** (Figure 17) could reduce greater than 50% of parasitemia after a 100 mg/kg oral treatment in mice infected with *Trypanosoma cruzi*. Molecular docking studies suggested that this compound could act as a trypanocidal agent by inhibiting cruzain, an essential enzyme for *Trypanosoma cruzi* metabolism that is typically inhibited by triazole compounds.

#### 2.4.5. Miscellaneous

**Figure 18.**
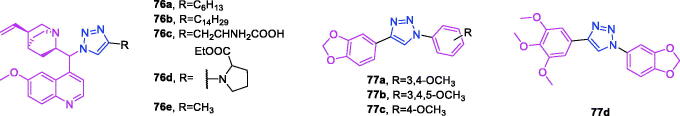
The chemical structures of anti-multiple parasitic compounds **76–77**.

Quinine-triazole derivatives not only possess antimalarial activity^139^, but also they exhibit antileishmanial activity^149^. Sahu et al.^149^ synthesised a series of quinine-triazole molecular hybrids. From the explored biological and toxicological evaluation it was observed that addition of triazole moiety to quinine resulted in reduction of toxicity of the conjugated scaffold. Out of the varied substituents lined to the parent quinine-triazole scaffold it was found that the tertiary amines linked compounds were relatively more potent than both the reference standards. On increasing the chain length of the compounds the potency of the compounds also exhibited an increasing trend. This may be attributed to the increased penetrating power rendered to the pharmacophoric lead due to long alkyl chain substitution of the parent scaffold. Among them, compounds **76a**, **76b**, **76c**, **76d**, and **76e** (Figure 18) possessed significant antimalarial (*Plasmodium falciparum*) and antileishmanial activities (*Leishmania donavani*), with IC_50_ values of 0.28, 0.28, 0.25, 0.33, 0.76 µM and 8.26, 4.4, 1.78, 3.95, and 4.06 µM, respectively. Further toxicological analyses established the median lethal dose (LD50), no observed adverse effect level (NOAEL), and human equivalent dose (HED) of the most potent compounds by acute and subacute toxicity studies performed in rodent animal models. The studies revealed that these compounds (**76a**, **76b**, **76c**, and **76d**) did not cause any measurable toxicity at a dose of 1000 mg/kg, and based on this, the corresponding HED was calculated to be 13.84 mg/kg.

Veraguensin (chemical formula: C_22_H_28_O_5_, molecular weight: 372.45, (2 *R*,3*S*,4*S*,5*S*)-2,5-Bis(3,4-dimethoxyphenyl)tetrahydro-3,4-dimethylfuran), grandisin (chemical formula: C_24_H_32_O_7_, molecular weight: 432.51, *rel*-(-)-(2*R*,3*R*,4*R*,5*R*)-Tetrahydro-3,4-dimethyl-2,5-bis(3,4,5-trimethoxyphenyl)furan), and machilin G (chemical formula: C_21_H_24_O_5_, molecular weight: 356.41, *rel*-(+)-5-[(2*R*,3*R*,4*S*,5*S*)-5–(3,4-Dimethoxyphenyl)tetrahydro-3,4-dimethyl-2-furanyl]-1,3-benzodioxole) are three of the sixteen 1,4-diaryl-1,2,3-triazole compounds derived from the natural products veraguensin, grandisin, and machilin G that were previously synthesised^150^. Biological activity tests against *L. amazonensis* promastigotes revealed that compounds **77a**, **77d**, and **77b** (Figure 18) were the most active, with maximum inhibitory concentration (IC_50_) values of 1.1, 3.71, and 7.23 µM that were higher than that of pentamidine (IC_50_ 8.9 µM). Compound **77a**, an analog of machilin G 3, was eightfold more active than was pentamidine, while **77d** and **77b** were 2.5- and 1.2-fold more active, respectively, than was pentamidine. Compounds **77a**, **77d**, and **77b** exhibited low cytotoxicity and possessed high selectivity indexes (SI) that are tens to hundreds of times higher than those of the recommended drugs for leishmaniasis, such as pentamidine and amphotericin B (SI 8.8 and 8.2, respectively) and for *Trypanosoma cruzi,* such as benznidazole (SI 13.2). Compound **77b** was highly active against *Leishmania infantum*, with an IC_50_ value of 5.2 µM, and derivative **77c** (Figure 18) exhibited an IC_50_ value of 28.6 µM against *Trypanosoma cruzi* trypomastigotes. Regarding SAR, hybrid 1,2,3-triazole compounds 77a and 77c and positional isomers 77 b and 77d, containing the methylenedioxy group present in machilin G, were the most active against the trypanosomatids, indicating that this group is responsible for the high antileishmanial activity and moderate antitrypanosomal activity of these compounds.

### 2.5. Antiviral activity

**Figure 19.**
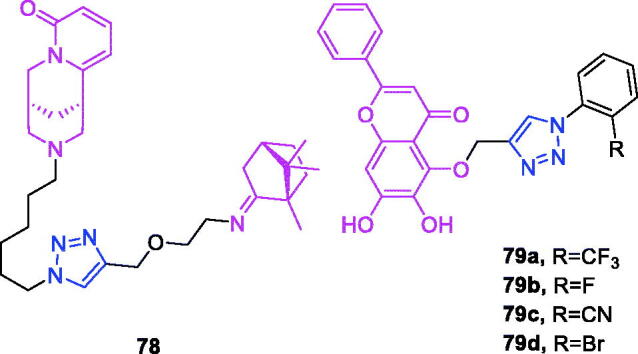
The chemical structures of antiviral compounds **78–79**.

Camphor (chemical formula: C_10_H_16_O, molecular weight: 152.23, 1,7,7-Trimethylbicyclo[2.2.1]heptan-2-one) is a member of the terpenoid family. Artyushin et al.^151^ synthesised six new terpene (+)-camphor and alkaloid (-)-cystine conjugates. Among them, imino-derivatives were, in general, of higher activity. Elongation of linker attached to the camphor moiety and introduction of bulky lipophilic groups decreased virusinhibiting activity. Conjugate **78** (Figure 19) that contains a cytisine fragment separated from the triazole ring by a –C_6_H_12_– aliphatic linker exhibited the highest activity at relatively low toxicity (values of 50% cytotoxic dose CC_50_=168 µmol, 50% inhibition dose IC_50_=8 µmol, selectivity index SI = 20). Its selectivity index appeared to be higher than that of the reference compound rimantadine.

Baicalein (chemical formula: C_15_H_10_O_5_, molecular weight: 270.24, 5,6,7-Trihydroxy-2-phenyl-4*H*-1-benzopyran-4-one) is an isolate of *Scutellaria baicalensis*. Baicalein triazole prevents respiratory tract infection by RSV through the suppression of oxidative damage^152^. The preventive effect of the most active compound (**79a**) (Figure 19) against RSV infection was studied in detail. Compound **79a** treatment increased IFN-β1 expression in BEAS-2B cells infected with RSV. Treatment of BEAS-2B cells with compound **79a** inhibited RSV-induced secretion of interleukin-6 and -8 cytokines. This compound also decreased RSV-induced nitric oxide and malondialdehyde production and inhibited the RSV-mediated activation of NF-κB, COX-2, Stat3, and MAPK. The p38 phosphorylation was enhanced significantly in RSV-infected cells in response to compound **79a** pre-treatment. RT-qPCR revealed that compound **79a** treatment of the RSV-infected mice significantly (*p* < 0.05) decreased viral load through reduction in the viral replication. In a mouse model of RSV-infection, compound **79a** treatment decreased interleukin-6, -8, and tumour necrosis factor-α expression. The levels of MPO, nitric oxide, and malondialdehyde were also decreased significantly by compound **79a** in the RSV infected mice BALF. This compound also reduced the infiltration of neutrophils and lymphocytes in the BALF of RVS-infected mice. In summary, compound **79a** inhibits RSV-infection and prevents pulmonary airway inflammation through the activation of the IFN signalling pathway. Structure activity relationship analysis revealed that all the four compounds **79a**, **79b**, **79c** and **79d** which demonstrated good activity against RSV induced infection contained substituents in the ortho-position. The substituents present in the ortho-position of compounds **79a**, **79b**, **79c** and **79d** are floro, trifloromethyl, nitrile and bromo, respectively. It appears that these compounds exhibit inhibitory effect on RSV infection by interacting with the proteins containing active site that can accommodate only ortho substituted compounds.

### 2.6. Antioxidant activity

**Figure 20.**
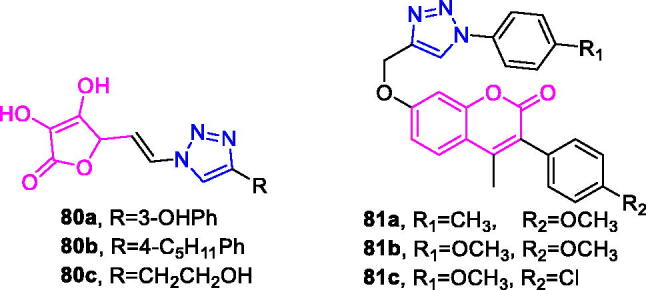
The chemical structures of antioxidant compounds **80–81**.

L-ascorbic acid (chemical formula: C_6_H_8_O_6_, molecular weight: 176.12, Vitamin C) exists in numerous types of fresh vegetables and fruits. The novel 4-substituted 1,2,3-triazole L-ascorbic acid conjugates possessing a hydroxyethylene spacer and their conformationally restricted 4,5-unsaturated analogs were synthesised as potential antioxidant agents by Harej and et al.^153^ An evaluation of the antioxidant activity of these novel compounds revealed that the majority of the 4,5-unsaturated L-ascorbic acid derivatives exhibited improved antioxidant activity compared to that of their saturated counterparts. *m*-Hydroxyphenyl (**80a**), p-pentylphenyl (**80b**), and 2-hydroxyethyl **(80c**) (Figure 20) substituted 4,5-unsaturated 1,2,3-triazole L-ascorbic acid derivatives exhibited highly efficient and rapid (within 5 min) 2,2-diphenyl-1-picrylhydrazyl (DPPH•) radical scavenging activity (**80a**, **80b**: IC_50_ = 0.06 mM; **80c**: IC_50_ = 0.07 mM). The structure activity analysis through principal component analysis indicated radical scavenging activity by the participation of OH group with favourable reaction parameters: the C3-OH group of saturated C4-C5(OH) derivatives and the C2-OH group of their unsaturated C4 = C5 analogues.

Coumarin-triazole derivatives not only possess anticancer activity^58^^,^^68^^,^^70^^,^^90–93^, anti-inflammatory activity^115^, antibacterial activity^128^^,^^129^, antifungal activity^133^, and antimalarial activity^140^, but also they exhibit antioxidant activity^154^. Coumarin-based 1,4-disubstituted 1,2,3-triazole derivatives were synthesised by Dharavath et al.^154^ All of the synthesised compounds were screened for their *in vitro* scavenging activity. The IC_50_ values ranged from 338.48 µM/mL to 0.064 µM/mL and varied widely compared to that of standard ascorbic acid (IC_50_ value 1.46 µM/mL). Compounds **81a** (IC_50_ value 0.061 µM/mL), **81b** (IC_50_ value 1.29 µM/mL), and **81c** (IC_50_ value 1.11 µM/mL) (Figure 20) possessed excellent activity, as the presence of methoxy and other electron-releasing groups such as methyl groups maintained the stability of the compounds and also contributed to the improved activity of compound **81a**. The presence of two methoxy groups in compound **81b** slightly decreased the activity. Further substitution of electron-withdrawing groups such as chlorine and methoxy in compound **81c** decreased the activity.

### 2.7. Anti-Alzheimer’s activity

**Figure 21.**
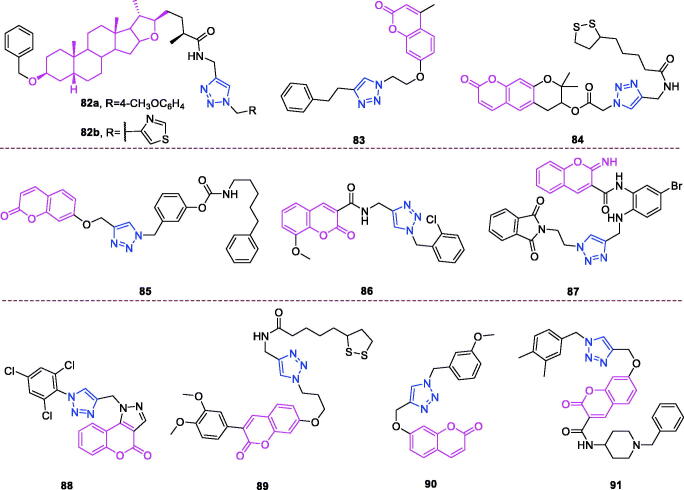
The chemical structures of anti-Alzheimer compounds **82–91**.

Sarsasapogenin (chemical formula: C_27_H_44_O_3_, molecular weight: 416.64, (3β,5β,25*S*)-Spirostan-3-ol) is an active ingredient in *Rhizoma anemarrhenae*. Wang et al.^155^ designed and synthesised a novel series of sarsasapogenin-triazolyl hybrids and evaluated their Aβ_1-42_ aggregation inhibitory activities. The result showed that the potent inhibitory activity exhibited by the benzyl groups series of compounds was possibly due to their steric effects and the ability of the benzyl groups forming pi-pi stacking interactions with the aromatic residues of Aβ. Among them, the most potent compound was **82a** (IC_50_ = 5.84 µM) (Figure 21), and this compound exhibited an inhibition ratio of 84.74%, which was approximately 1.5- and 5.2-fold higher than that of curcumin (55.87%). Compound **82b** (IC_50_ = 8.28 µM) (Figure 21) containing a thiazole moiety also exhibited good Aβ_1-42_ aggregation inhibitory activity that was improved compared to that of the standard drug curcumin (IC_50_ = 14.99 µM). Moreover, **82a** and **82b** exhibited moderate neuroprotective effects against H_2_O_2_-induced neurotoxicity in SH-SY5Y cells. Oral treatment with **82a** and **82b** significantly ameliorated cognitive impairments in behavioural tests, and TUNEL staining revealed that **82a** and **82b** attenuated neuronal loss in the brain.

Coumarin-triazole derivatives not only possess anticancer activity^58^^,^^68^^,^^70^^,^^90–93^, anti-inflammatory activity^115^, antibacterial activity^128^^,^^129^, antifungal activity^133^, antimalarial activity^140^, and antioxidant activity^154^, but also they exhibit anti-Alzheimer’s activity^156–164^. In 2016, Torres et al.^156^ screened the *in vitro* acetylcholinesterase inhibitor activity of a novel series of 1,2,3-triazole-linked coumarin hybrids. The most prominent compound was coumarin **83** (triazole and 4-methylcoumarins) (Figure 21), and this compound inhibited nearly 60% of the acetylcholinesterase activity at a concentration of 200 µmol/L. However, docking simulations suggest that this compound binds similarly to donepezil and, consequently, this compound can putatively block the secondary non-cholinergic functions of the enzyme, including adhesion, differentiation, and deposition of beta-amyloid in Alzheimer’s disease. Therefore, can be explored in 4-methylcoumarins/1,2,3-triazoles conjugates to yield multitarget ligands in the search of new compounds for the treatment of Alzheimer’s disease. The same year, Park et al.^157^ designed and synthesised new triazole-linked decursinol derivatives possessing potent inhibitory activities against cholinesterase (acetylcholinesterase [AChE] and butyrylcholinesterase [BuChE]). Compound **84** (IC_50_ = 5.89 ± 0.31 mM against BuChE) (Figure 21) possessed more effective inhibitory activity against BuChE than did galantamine (IC_50_ = 9.4 ± 2.5 mM). Moreover, compound **84** exhibited no inhibitory activity against AChE (IC_50_ value > 350 mM). Inhibitory activity and selectivity (AchE/BuChE) of triazole linked decursinol derivatives may result from not decursinol or triazolee moiety but hybrid compounds. Since decursinol itself is an interesting bioactive pharmacological compound, the new biological activity of decursinol derivatives against BuChE will result in beneficial effects for treating AD patients. Also, selective inhibition of BuChE over AChE may have another beneficial effect compared with exclusive use of AChE inhibitors. Additionally, in the same year, the multi-target-directed ligand paradigm was applied to the design of carbamates able to simultaneously target the recently proposed endocannabinoid system and the classic cholinesterase system to achieve effective dual FAAH/cholinesterase inhibitors^158^. Among the two series of synthesised compounds, some derivatives proved to be extremely potent on a single target, and compound **85** (Figure 21) was identified as an effective dual FAAH/ChE inhibitor, with well-balanced nanomolar activities (IC_50_ = 42.7 nM and 27.9 nM, respectively), and substitution at the phenyl ring carrying the carbamate group proved to be crucial for structure-activity relationships. Thus, compound **85** may be considered as a new promising candidate for AD treatment. In 2017, a novel series of chromenones linked to the 1,2,3-triazole ring system were synthesised and evaluated for their anti-AChE activity by Akbarzadeh et al.^159^ The presence of halogen at 2-position of aryl group connected to 1,2,3-triazole increased AChEI activity. Among them, *N*-((1–(2-chlorobenzyl)-1*H*-1,2,3-triazol-5-yl)methyl)-8-methoxy-2-oxo-2*H*-chromene-3-carboxamide (**86**) (Figure 21) bearing methoxy group on the chromenone moiety and 2-chlorophenyl on the pendant 1,2,3-triazole group, exhibited good anti-acetylcholinesterase activity (IC_50_=15.42 µM). Additionally, compound **86** demonstrated a neuroprotective effect against H_2_O_2_-induced cell death in PC12 neurons; however, it exhibited no beta-secretase (BACE1) inhibitory activity. Docking and kinetic studies separately confirmed the dual binding activity of compound **86**, as it targeted both the catalytic active site (CAS) and the peripheral anionic site (PAS) of AChE. In the same year, multifunctional iminochromene-2*H*-carboxamide derivatives containing different aminomethylene triazoles with BACE1 inhibitory, neuroprotective, and metal chelating properties targeting Alzheimer’s disease were synthesised^160^. The majority of the synthesised compounds were demonstrated to possess moderate to potent BACE1 inhibitory activity according to a FRET assay. Substitution of phthalimide on amino methylene triazole plays a significant role in BACE1 inhibition. Compound **87** (Figure 21) that possesses a phthalimide pendant on the amino methylene triazole was the most potent derivative, with an IC_50_ value of 2.2 µM. Compound **87** did not exhibit any cytotoxicity up to a concentration of 100 µM. In 2018, Gharbi et al.^161^ studied the anticholinesterase and cytotoxic activities of 2-((1–(2,4,6-trichlorophenyl)-1H-1,2,3-triazol-4-yl)methyl) benzopyrano[4,3-*c*] pyrazol-4(2*H*)-one. The result showed the importance of the triazole ring to enhance the activity. The importance of the chlorine atom, characterised by an electro-attractor effect, compared to the methyl group which, on the contrary, has an electro-donor effect. The importance of the position of the triazole ring in the molecule,and the position of this system on the nitrogen N-1 seems more favourable to this activity. Compound **88** (IC_50_ = 18 µM) (Figure 21) was found to possess the most potent anti-cholinesterase activity and to be less cytotoxic (IC_50_ = 6.0 µM against the HCT-116 cell line). Compound **88** was the starting compound used to treat the multi-targeted anticholinesterases, anti-5-lipoxygenases, and anti-tyrosinases and to assess anti-cancer activities. In the same year, thirty coumarin-lipoic acid conjugates were synthesised and screened for their new multi-target-directed ligands (MTDLs) for the treatment of Alzheimer’s disease by Jalili-Baleh et al.^162^. Among them, 3,4-dimethoxyphenyl-coumarin derivative **89** (IC_50_ = 16.4 µM) (Figure 21) was approximately twofold more effective than the reference drug donepezil in terms of the inhibitory activity on self-induced and AChE-induced Aβ_1-42_ aggregation. Moreover, compound **89** provided significant protection against Aβ_1-42_-induced cytotoxicity that was superior to that of donepezil. The compound **89** contains a propylene liker (*n* = 3). Elongation of linker (*n* = 4 and 5) led to homologs with no activity on cholinesterases. On the other hand, the 3,4-dichlorophenyl-coumarin derivatives with 4 C or 5 C linker showed suitable inhibitory activity against BuChE (IC_50_ values of 10.3 and 7.8 µM, respectively). The 3,4-dichlorophenyl-coumarin analog with propylene spacer (*n* = 3) showed marginal activity against BuChE (IC_50_ = 73.5 µM). Therefore, it can be concluded that in 3,4-dichlorophenyl-coumarin derivatives, the elongation of linker increased the anti-BuChE potency. Additionally, a novel hybrid series of umbellipherone and benzyl amine scaffolds linked via triazole rings was synthesised and evaluated for use as both an acetylcholinesterase (AChE) and butyrylcholinesterase (BuChE) inhibitor^163^. All of the synthesised compounds possessed moderate to excellent inhibitory activities. In umbelliferone series, substitution at C-2 position of the phenyl ring was shown to increase the activity in comparison with the unsubstituted derivatives. The order of activity was Cl > F > NO_2_ > H, which shows that hydrophobic, bulky and electron withdrawing substituent at C-2 position is needed for optimal activity. According to the IC_50_s in umbellipherone series, the presence of polar substituents such as methoxy at 3 position afforded compounds, exhibiting potent AChE inhibition OCH_3_ > H > C l > F > Br = CH_3_. Of these, compound **90** (Figure 21) that possessed a 3-methoxy substituent on the benzyl moiety was the most active (AChE and BuChE, IC_50_ = 3.4 and 1.1 µM, respectively). Neuroprotection evaluation revealed that this compound efficiently protected PC_12_ neurons against H_2_O_2_-induced cell death. In 2019, a set of novel 1,2,3-triazole-chromenone carboxamide derivatives was synthesised and screened for *in vitro* cholinesterase inhibitory activity by Mina Saeedi^164^. The majority of the synthesised compounds were inactive at a concentration of 100 µM, whereas *N*-(1-benzylpiperidin-4-yl)-7-((1–(3,4-dimethylbenzyl)-1*H*-1,2,3-triazol-4-yl)methoxy)-2-oxo-2*H*-chromene-3-carboxamide (**91**) (Figure 21) displayed potent acetylcholinesterase inhibitory activity (IC_50_ = 1.80 µM), although it was inactive towards butyrylcholinesterase (IC_50_ ≥ 100 µM). The compound **91** possessed benzylpiperidinyl moiety connected to amide functional group and 3,4-dimethylbenzyl connected to 1,2,3-traizole moiety. Compound **91** was evaluated for its BACE1 inhibitory activity, and the calculated IC_50_ of 21.13 µM confirmed the desired inhibitory activity. This compound also exhibited a satisfactory neuroprotective effect against H_2_O_2_-induced cell death in PC12 neurons at 50 µM as well as metal chelating ability towards Fe^2+^, Cu^2+^, and Zn^2+^ ions. It seems that benzylpiperidinyl moiety induced better anti-AChE activity than 3-morpholinopropyl which can be associated to similar moiety (benzylpiperidinyl group) ubiquitous in donepezil. Also, the presence of benzylpiperidinyl moiety and 3,4-disubstituted benzyl group connected to 1,2,3-triazole ring is significant for induction of AChEI activity in this series of 1,2,3-triazole-chromenone carboxamides.

### 2.8. Enzyme inhibitors

#### 2.8.1. Pancreatic lipase inhibitors

**Figure 22.**
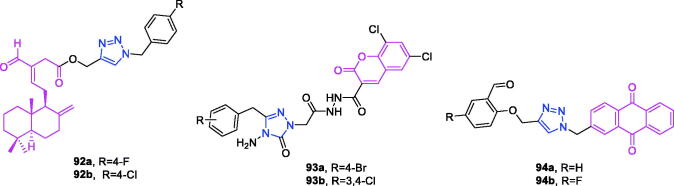
The chemical structures of enzyme inhibitors **92–94**.

(*E*)-labda 8(17), 12-diene-15, 16-dial (Chemical formula: C_20_H_30_O_2_, molecular weight: 302.4580) is a labdane that was isolated from the fresh rhizomes of *Curcuma amada*^165^. Jalaja et al.^166^ designed and synthesised a new natural product-derived labdane incorporating appended triazoles with pancreatic lipase inhibition potential. The cytotoxicity of the compounds against the Hep G2 human liver carcinoma-derived cell line was measured according to MTT assays. Based on the percentage of cell viability, none of the compounds exhibited any signs of toxicity at any of the tested concentrations. Among the semisynthetic derivatives, the labdane traizole appendages **92a** and **92b** (Figure 22) were the most active candidates from the series and exhibited excellent pancreatic lipase inhibitory activity (IC_50_: 0.75 ± 0.02 µM and 0.77 ± 0.01 µM) that was slightly higher than that of the positive control Orlistat (IC_50_: 0.8 ± 0.03 µM). In a structure activity relationship point of view, we observed that the labdane-triazole hybrids incorporating benzyl azides were more active than the phenacyl azides. Most of the analogues synthesised from benzyl azides exhibited excellent inhibition property slightly better than or equal to that of Orlistat. In contrast, the triazole analogues synthesised from phenacyl azides did not show any significant inhibition potential except for the compound 6 m. In precise, among the triazoles incorporated from the variously substituted benzyl azides, all the para-substituted analogues showed the lowest IC_50_. However, the unsubstituted, *ortho*- and *meta*-substituted benzyl azide incorporated triazole appendages showed moderate activity. Interestingly, there was no clear trend followed by the nature of the para substitution, i.e. among the halogen, electron donating and electron withdrawing groups. Overall, among the various substituted hybrids **92a** and **92b** with *p*-F and *p*-Cl substituted benzyl azide incorporated triazole appendages were found to be the most potent candidates of the series.

Coumarin-triazole derivatives not only possess anticancer activity^58^^,^^68^^,^^70^^,^^90–93^, anti-inflammatory activity^115^, antibacterial activity^128^^,^^129^, antifungal activity^133^, antimalarial activity^140^, antioxidant activity^154^, and anti-Alzheimer’s activity^156–164^, but also they exhibit pancrelipase inhibitory activity^167^. Kahveci et al.^167^ designed, synthesised, and studied the anti-lipase activity of the coumarin-triazole hybrid molecule. The majority of the compounds possessed anti-lipase activities at various concentrations. Among the tested compounds, **93a** and **93b** (Figure 22) exhibited the best anti-lipase activity. These compounds inhibited pancreatic lipase by 99.30 ± 0.56% and 99.85 ± 1.21%, respectively, at a concentration of 10 µM. Orlistat, a pancreatic lipase inhibitor used as an anti-obesity drug, yielded an inhibitory effect of 99.88 ± 0.43% at a concentration of 300 nM (IC_50_ = 0.41 ± 0.01 nM). The IC_50_ values of compounds **93a** and **93b** were calculated as 2.64 ± 0.33 µM and 1.80 ± 0.08 µM, respectively. Based on the biological activity, these results indicate that coumarin-triazol hybrid molecules show more activity than coumarin and triazol derivatives.

#### 2.8.2. Xanthine oxidase inhibitors

Anthraquinone (chemical formula: C_14_H_8_O_2_, molecular weight: 208.21, 9,10-Anthracenedione) possesses the structural core of anthracyclines. (1*H*-1,2,3-triazol-4-yl)methoxybenzaldehyde derivatives containing an anthraquinone moiety were synthesised and identified as novel xanthine oxidase inhibitors^168^. Among them, the most promising compounds (**94a** [IC_50_ = 0.6 µM] and **94b** [IC_50_ = 0.8 µM]) (Figure 22) were obtained, and these compounds exhibited greater than 10-fold higher potencies compared to that of the reference xanthine oxidase inhibitor allopurinol. The structure activity relationship analysis revealed that the benzaldehyde moiety played a more important role than did the anthraquinone moiety in its inhibitory potency. Additionally, a formyl group fixed at the 2-position of the phenyl moiety was essential for bioactivity. The Lineweaver–Burk plot showed that compound **94a** acted as a mixed-type xanthine oxidase inhibitor. The basis for the significant inhibition of xanthine oxidase by compound **94a** was rationalised by molecular modelling studies.

## 3. Conclusion

Natural products or their derivatives are increasingly used in commercial drugs, which reflects their widespread use as lead compounds for discovering new drugs with novel structures and mechanisms. Triazole has enough potential therapeutic applicability, and is still expanding, it is a multifunctional stent in medicinal chemistry. This review article is an effort to summarise medicinal chemistry investigations of natural product-triazole derivatives in the past six years, in search for new natural product-triazole derivatives which may be an excellent source of promising biological activities. It will help the scientific community rationally design and develop varied, optimised, novel and target oriented natural product-triazole based drugs for treating multifactorial diseases. The structural analysis, molecular docking, activity analysis and mechanism research may provide convenience for further exploration and development of new natural product-triazole derivatives with improved efficacy and reduced toxicity.

## 4. Future perspective

Due to its wide chemical diversity, natural products have become an important source of biologically active compounds. However, natural biologically active compounds may have inappropriate pharmacological properties, limiting their use, such as cytotoxicity, high lipophilicity or poor oral absorption. The inability to obtain most of these derivatives from sustainable sources is another major limitation in the use of natural products in drug development. Therefore, natural products that have undergone structural modifications to facilitate the discovery of new drugs with novel structures and mechanisms are widely used as lead compounds. The triazole part has developed into an indispensable heterocyclic scaffold due to its extensive biological properties, especially for the production of natural product-triazole hybrids, even though the triazole part does not exist in nature. This review article mainly studies the biological activity and structure of natural product-triazole derivatives. Among the many biological activities, we comprehensively reviewed the progress in many aspects of the natural product triazole derivatives that have been specially reported in the past six years, such as anti-cancer, anti-inflammatory, anti-microbial, anti-parasitic, anti-viral, antioxidant, and anti-Alzheimer’s disease and enzyme inhibitor action. In this review, we aimed to provide a wide range of data resources on natural product triazole derivatives to medicinal chemists who are engaged in drug design and development, so as to help them conduct more fertile and organised drug discovery operation in the process of experimental studies. Simple structural analysis and molecular docking would be helpful in the structural modification of natural product derivatives with triazole scaffolds. Activity analysis and mechanism research would be very useful for enhancing pharmacokinetic properties and biological activity, and overcoming the difficulty of reducing toxicity and increasing selectivity.

## References

[1] Hamilton GR, Baskett TF. In the arms of Morpheus the development of morphine for postoperative pain relief. Can J Anaesth 2000;47:367–74. 10764185 10.1007/BF03020955

[2] ] Cragg GM, Newman DJ, Snader KM. Natural products in drug discovery and development. J Nat Prod 1997;60:52–60. 9014353 10.1021/np9604893

[3] Newman DJ, Cragg GM, Snader KM. Natural products as sources of new drugs over the period 1981-2002. J Nat Prod 2003;66:1022–37. 12880330 10.1021/np030096l

[4] Newman DJ, Cragg GM. Natural products as sources of new drugs over the last 25 years. J Nat Prod 2007;70:461–77. 17309302 10.1021/np068054v

[5] Newman DJ, Cragg GM. Natural products as sources of new drugs over the 30 years from 1981 to 2010. J Nat Prod 2012;75:311–35. 22316239 10.1021/np200906s PMC3721181

[6] Newman DJ, Cragg GM. Natural products as sources of new drugs from 1981 to 2014. J Nat Prod 2016;79:629–61. 26852623 10.1021/acs.jnatprod.5b01055

[7] Newman DJ, Giddings LA. Natural products as leads to antitumor drugs. Phytochem Rev 2014;13:123–37.

[8] Cragg GM, Newman DJ. Natural products: a continuing source of novel drug leads. Biochim Biophys Acta 2013;1830:3670–95. 23428572 10.1016/j.bbagen.2013.02.008 PMC3672862

[9] Aromi G, Barrios LA, Roubeau O, Gamez P. Triazoles and tetrazoles: prime ligands to generate remarkable coordination materials. Coordin Chem Rev 2011;255:485–546.

[10] Bonandi E, Christodoulou MS, Fumagalli G, et al. The 1,2,3-triazole ring as a bioisostere in medicinal chemistry. Drug Discovery Today 2017;22:1572–81. 28676407 10.1016/j.drudis.2017.05.014

[11] Kaur P, Chawla A. 1,2,4-Triazole: a review of pharmacological activities. Int Res J Pharm 2017;8:10–29.

[12] Kapron B, Luszczki JJ, Plazinska A, et al. Development of the 1,2,4-triazole-based anticonvulsant drug candidates acting on the voltage-gated sodium channels. Insights from in-vivo, in-vitro, and in-silico studies. Eur J Pharm Sci 2019;129:42–57. 30594731 10.1016/j.ejps.2018.12.018

[13] Konwar M, Ali AA, Chetia M, et al. Fehling solution/DIPEA/hydrazine: an alternative catalytic medium for regioselective synthesis of 1,4-disubstituted-1H-1,2,3-triazoles using azide–alkyne cycloaddition reaction. Tetrahedron Lett 2016;57:4473–6.

[14] Zhang GR, Ren Y, Yin XM, Quan ZS. Synthesis and evaluation of the anticonvulsant activities of new 5-substitued-[1,2,4]triazolo[4,3-a]quinoxalin-4(5H)-one derivatives. Lett Drug Des Discov 2018;15:406–13.

[15] Liu XJ, Zhang HJ, Quan ZS. Synthesis and evaluation of the anticonvulsant activities of 2,3-dihydrophthalazine-1,4-dione derivatives. Med Chem Res 2017;26:1935–46.

[16] Ren Y, Shen QK, Ding MM, et al. Synthesis and anticonvulsant activities of 4-alkoxyl-[1,2,4]triazolo[4,3-a]quinoxaline derivatives. Lat Am J Pharm 2016;35:2169–75.

[17] Liu CF, Zhang HJ, Quan ZS. Synthesis and anticonvulsant activity of novel 3-(2(4H-1,2,4-triazol-4-yl)ethyl)-1-alkyl-1H-indole derivatives. Lett Drug Des Discov. 2016;13:833–9.

[18] Zhang HJ, Wang SB, Wen X, et al. Design, synthesis, and evaluation of the anticonvulsant and antidepressant activities of pyrido[2,3-d]pyrimidine derivatives. Med Chem Res 2016;25:1287–98.

[19] Valdomir G, Fernández MÁ, Lagunes I, et al. Oxa/thiazole-tetrahydropyran triazole-linked hybrids with selective antiproliferative activity against human tumour cells. New J Chem 2018;42:13784–9.

[20] Fan YL, Ke X, Liu M. Coumarin-triazole hybrids and their biological activities. J Heterocyclic Chem 2018;55:791–802.

[21] Akhtar J, Khan AA, Ali Z, et al. Structure-activity relationship (SAR) study and design strategies of nitrogen-containing heterocyclic moieties for their anticancer activities. Eur J Med Chem 2017;125:143–89. 27662031 10.1016/j.ejmech.2016.09.023

[22] Kommidi H, Guo H, Nurili F, et al. (18)F-Positron emitting/trimethine cyanine-fluorescent contrast for image-guided prostate cancer management. J Med Chem 2018;61:4256–62. 29676909 10.1021/acs.jmedchem.8b00240 PMC6263152

[23] Huang S, Han Y, Chen M, et al. Radiosynthesis and biological evaluation of 18F-labeled 4-anilinoquinazoline derivative (18F-FEA-Erlotinib) as a potential EGFR PET agent. Bioorg Med Chem Lett 2018;28:1143–8. 29486966 10.1016/j.bmcl.2017.08.066

[24] He YW, Dong CZ, Zhao JY, et al. 1,2,3-Triazole-containing derivatives of rupestonic acid: click-chemical synthesis and antiviral activities against influenza viruses. Eur J Med Chem 2014;76:245–55. 24583605 10.1016/j.ejmech.2014.02.029

[25] Xu Z, Song XF, Hu YQ, Qiang M, et al. Azide-alkyne cycloaddition towards 1H-1,2,3-triazole-tethered gatifloxacin and isatin conjugates: design, synthesis and in vitro anti-mycobacterial evaluation. Eur J Med Chem 2017;138:66–71. 28646656 10.1016/j.ejmech.2017.05.057

[26] García-Vanegas JJ, Ramírez-Villalva A, Fuentes-Benites A, et al. Synthesis and in-vitro biological evaluation of 1,1-diaryl-2-(1,2,3)triazol-1-yl-ethanol derivatives as antifungal compounds flutriafol analogues. J Chem Sci 2019;131:27.

[27] Kaushik CP, Luxmi R, Kumar A, et al. Antibacterial evaluation and QSAR studies of 1,2,3-triazole bridged with amide functionalities. Indian J Chem B 2019;58:88–95.

[28] Satheeshkumar C, Ravivarma M, Arjun P, et al. Synthesis, anti-microbial activity and molecular docking studies on triazolylcoumarin derivatives. J Chem Sci 2015;127:565–74.

[29] Zhou J, Stapleton P, Haider S, Healy J. Boronic acid inhibitors of the class A β-lactamase KPC-2. Bioorg Med Chem 2018;26:2921–7. 29784271 10.1016/j.bmc.2018.04.055

[30] Lopes SMM, Novais JS, Costa DCS, et al. Hetero-Diels-Alder reactions of novel 3-triazolyl-nitrosoalkenes as an approach to functionalized 1,2,3-triazoles with antibacterial profile. Eur J Med Chem 2018;143:1010–20. 29232578 10.1016/j.ejmech.2017.11.052

[31] Fletcher JT, Sobczyk JM, Gwazdacz SC, Blanck AJ. Antimicrobial 1,3,4-trisubstituted-1,2,3-triazolium salts. Bioorg Med Chem Lett 2018;28:3320–3. 30219525 10.1016/j.bmcl.2018.09.011 PMC6214158

[32] Rezaei-Matehkolaei A, Khodavaisy S, Alshahni MM, et al. In vitro antifungal activity of novel triazole efinaconazole and five comparators against dermatophyte isolates. Antimicrob Agents Chemother 2018;62:e02423–02417. 29530856 10.1128/AAC.02423-17 PMC5923135

[33] Zhang S, Xu Z, Gao C, et al. Triazole derivatives and their anti-tubercular activity. Eur J Med Chem 2017;138:501–13. 28692915 10.1016/j.ejmech.2017.06.051

[34] Moghimi S, Goli-Garmroodi F, Pilali H, et al. Synthesis and anti-acetylcholinesterase activity of benzotriazinone-triazole systems. Journal of Chemical Sciences 2016;128:1445–9.

[35] Pan FJ, Wang SB, Liu DC, et al. Synthesis of 4-phenylthieno[2,3-e][1,2,4]triazolo[4,3-a]pyrimidine-5(4H)-one derivatives and evaluation of their anti-inflammatory activity. Lett Drug Des Discov 2016;13:141–8.

[36] Angajala KK, Vianala S, Macha R, et al. Synthesis, anti-inflammatory, bactericidal activities and docking studies of novel 1,2,3-triazoles derived from ibuprofen using click chemistry. Springerplus 2016;5:423. 27104111 10.1186/s40064-016-2052-5 PMC4828371

[37] Mady MF, Awad GE, Jorgensen KB. Ultrasound-assisted synthesis of novel 1,2,3-triazoles coupled diaryl sulfone moieties by the CuAAC reaction, and biological evaluation of them as antioxidant and antimicrobial agents. Eur J Med Chem 2014;84:433–43. 25038485 10.1016/j.ejmech.2014.07.042

[38] Rajavelu K, Subaraja M, Rajakumar P. Synthesis, optical properties, and antioxidant and anticancer activity of benzoheterazole dendrimers with triazole bridging unit. New Journal of Chemistry 2018;42:3282–92.

[39] Kumar KA, Kalluraya B, Kumar SM. Synthesis and in-vitro antioxidant activities of some coumarin derivatives containing 1,2,3-triazole ring. Phosphorus Sulfur 2018;193:294–9.

[40] Santosh R, Selvam MK, Kanekar SU, Nagaraja GK. Synthesis, characterization, antibacterial and antioxidant studies of some heterocyclic compounds from triazole‐linked chalcone derivatives. ChemistrySelect 2018;3:6338–43.

[41] Batra N, Rajendran V, Agarwal D, et al. Synthesis and antimalarial evaluation of [1,2,3]-triazole-tethered sulfonamide-berberine hybrids. Chemistryselect 2018;3:9790–3.

[42] Roy KK. Targeting the active sites of malarial proteases for antimalarial drug discovery: approaches, progress and challenges. Int J Antimicrob Agents 2017;50:287–302. 28668681 10.1016/j.ijantimicag.2017.04.006

[43] Fan YL, Cheng XW, Wu JB, et al. Antiplasmodial and antimalarial activities of quinolone derivatives: an overview. Eur J Med Chem 2018;146:1–14. 29360043 10.1016/j.ejmech.2018.01.039

[44] Chinthala Y, Thakur S, Tirunagari S, et al. Synthesis, docking and ADMET studies of novel chalcone triazoles for anti-cancer and anti-diabetic activity. Eur J Med Chem 2015;93:564–73. 25743216 10.1016/j.ejmech.2015.02.027

[45] Xu JH, Fan YL, Zhou J. Quinolone-triazole hybrids and their biological activities. J Heterocyclic Chem 2018;55:1854–62.

[46] Sheng C, Zhang W. New lead structures in antifungal drug discovery. Curr Med Chem 2011;18:733–66. 21182484 10.2174/092986711794480113

[47] Neu HC, Fu KP. Cefatrizine activity compared with that of other cephalosporins. Antimicrob Agents Chemother 1979;15:209–12. 426514 10.1128/aac.15.2.209 PMC352634

[48] Soltis MJ, Yeh HJ, Cole KA, et al. Identification and characterization of human metabolites of CAI [5-amino-1-1(4'-chlorobenzoyl-3,5-dichlorobenzyl)-1,2,3-triazole- 4-carboxamide). Drug Metab Dispos 1996;24:799–806. 8818579

[49] Higashitani F, Hyodo A, Ishida N, et al. Inhibition of beta-lactamases by tazobactam and in-vitro antibacterial activity of tazobactam combined with piperacillin. J Antimicrob Chemother 1990;25:567–74. 2161820 10.1093/jac/25.4.567

[50] Zhao L, Mao L, Hong G, et al. Design, synthesis and anticancer activity of matrine-1H-1,2,3-triazole-chalcone conjugates. Bioorg Med Chem Lett 2015;25:2540–4. 25959813 10.1016/j.bmcl.2015.04.051

[51] Huang RZ, Liang GB, Li MS, et al. Synthesis and discovery of asiatic acid based 1,2,3-triazole derivatives as antitumor agents blocking NF-κB activation and cell migration. MedChemComm 2019;10:584–97. 31057738 10.1039/c8md00620b PMC6484948

[52] El-Seedi HR, El-Barbary MA, El-Ghorab DMH, et al. Recent insights into the biosynthesis and biological activities of natural xanthones. Curr Med Chem 2010;17:854–901. 20156171 10.2174/092986710790712147

[53] Wu JQ, Dai JW, Zhang YY, et al. Synthesis of novel xanthone analogues and their growth inhibitory activity against human lung cancer A549 cells. Drug Des Devel Ther 2019;13:4239–46. 10.2147/DDDT.S217827 PMC6916687 31853172

[54] Linseisen J, Radtke J, Wolfram G. [Flavonoid intake of adults in a Bavarian subgroup of the national food consumption survey]. Z Ernahrungswiss 1997;36:403–12. 9467240 10.1007/BF01617836

[55] Gutam M, Mokenapelli S, Yerrabelli JR, et al. Synthesis and cytotoxicity of novel (E)-2-phenylchroman-4-one-O-((1-substituted-1H-1,2,3-triazol-4-yl)methyl) oxime derivatives. Synth Commun 2020;50:1883–91.

[56] Wall ME, Wani MC, Cook CE, et al. Plant antitumor agents. I. The isolation and structure of camptothecin, a novel alkaloidal leukemia and tumor inhibitor from *Camptotheca acuminata*. J Am Chem 1966;88:3888–90.

[57] Xu XG, Wu YL, Liu WF, et al. Discovery of 7-methyl-10-hydroxyhomocamptothecins with 1,2,3-triazole moiety as potent topoisomerase I inhibitors. Chem Biol Drug Des 2016;88:398–403. 27062430 10.1111/cbdd.12767

[58] Kumari P, Dubey S, Venkatachalapathy S, et al. Synthesis of new triazole linked carbohybrids with ROS-mediated toxicity in breast cancer. New J Chem 2019;43:18590–600.

[59] Hou W, Luo Z, Zhang G, et al. Click chemistry-based synthesis and anticancer activity evaluation of novel C-14 1,2,3-triazole dehydroabietic acid hybrids. Eur J Med Chem 2017;138:1042–52. 28759877 10.1016/j.ejmech.2017.07.049

[60] Sharma B, Gu L, Pillay RP, et al. Design, synthesis, and anti-proliferative evaluation of 1H-1,2,3-triazole grafted tetrahydro-β-carboline-chalcone/ferrocenylchalcone conjugates in estrogen responsive and triple negative breast cancer cells. New J Chem 2020;44:11137–47.

[61] Gurrapu N, Praveen Kumar E, Kolluri PK, et al. Synthesis, biological evaluation and molecular docking studies of novel 1,2,3-triazole tethered chalcone hybrids as potential anticancer agents. J Mol Struct 2020;1217:128356.

[62] Seo CS, Lim HS, Jeong SJ, Shin HK. Anti-allergic effects of sesquiterpene lactones from the root of Aucklandia lappa Decne. Mol Med Rep 2015;12:7789–95. 26398906 10.3892/mmr.2015.4342

[63] Pavan Kumar C, Devi A, Ashok Yadav P, et al. "Click" reaction mediated synthesis of costunolide and dehydrocostuslactone derivatives and evaluation of their cytotoxic activity. J Asian Nat Prod Res 2016;18:1063–78. 27329166 10.1080/10286020.2016.1193012

[64] Ottoni FM, Gomes ER, Padua RM, et al. Synthesis and cytotoxicity evaluation of glycosidic derivatives of lawsone against breast cancer cell lines. Bioorg Med Chem Lett 2020;30:126817. 31810778 10.1016/j.bmcl.2019.126817

[65] Zhang HJ, Zhang GR, Piao HR, Quan ZS. Synthesis and characterisation of celastrol derivatives as potential anticancer agents. J Enzyme Inhib Med Chem 2017;33:190–8. 29231066 10.1080/14756366.2017.1404590 PMC6009949

[66] Shen QK, Liu CF, Zhang HJ, et al. Design and synthesis of new triazoles linked to xanthotoxin for potent and highly selective anti-gastric cancer agents. Bioorg Med Chem Lett 2017;27:4871–5. 28947149 10.1016/j.bmcl.2017.09.040

[67] Qi Y, Ding Z, Yao Y, et al. Novel triazole analogs of apigenin-7-methyl ether exhibit potent antitumor activity against ovarian carcinoma cells via the induction of mitochondrial-mediated apoptosis. Exp Ther Med 2019;17:1670–6. 30783435 10.3892/etm.2018.7138 PMC6364180

[68] Kraljevic TG, Harej A, Sedic M, et al. Synthesis, in vitro anticancer and antibacterial activities and in silico studies of new 4-substituted 1,2,3-triazole-coumarin hybrids. Eur J Med Chem 2016;124:794–808. 27639370 10.1016/j.ejmech.2016.08.062

[69] Li FY, Huang L, Li Q, et al. Synthesis and antiproliferative evaluation of novel hybrids of dehydroabietic acid bearing 1,2,3-triazole moiety. Molecules 2019;24:4191. 10.3390/molecules24224191 PMC6891475 31752282

[70] Farley CM, Dibwe DF, Ueda JY, et al. Evaluation of synthetic coumarins for antiausterity cytotoxicity against pancreatic cancers. Bioorg Med Chem Lett 2016;26:1471–4. 26832787 10.1016/j.bmcl.2016.01.054

[71] Mosettig E, Beglinger U, Dolder F, et al. The absolute configuration of steviol and isosteviol. J Am Chem Soc 1963;85:2305–9.

[72] Avent AG, Hanson JR, De Oliveira BH. Hydrolysis of the diterpenoid glycoside, stevioside. Phytochemistry 1990;29:2712–5.

[73] Liu CJ, Liu YP, Yu SL, et al. Syntheses, cytotoxic activity evaluation and HQSAR study of 1,2,3-triazole-linked isosteviol derivatives as potential anticancer agents. Bioorg Med Chem Lett 2016;26:5455–61. 27777008 10.1016/j.bmcl.2016.10.028

[74] Pettit GR, Singh SB, Niven ML, et al. Isolation, structure, and synthesis of combretastatins A-1 and B-1, potent new inhibitors of microtubule assembly, derived from *Combretum caffrum*. J Nat Prod 1987;50:119–31. 3598594 10.1021/np50049a016

[75] Pettit GR, Singh SB, Hamel E, et al. Isolation and structure of the strong cell growth and tubulin inhibitor combretastatin A-4. Experientia 1989;45:209–11. 2920809 10.1007/BF01954881

[76] Li YH, Zhang B, Yang HK, et al. Design, synthesis, and biological evaluation of novel alkylsulfanyl-1,2,4-triazoles as cis-restricted combretastatin A-4 analogues. Eur J Med Chem 2017;125:1098–106. 27810596 10.1016/j.ejmech.2016.10.051

[77] Alakurtti S, Makela T, Koskimies S, Yli-Kauhaluoma J. Pharmacological properties of the ubiquitous natural product betulin. Eur J Pharm Sci 2006;29:1–13. 16716572 10.1016/j.ejps.2006.04.006

[78] Cichewicz RH, Kouzi SA. Chemistry, biological activity, and chemotherapeutic potential of betulinic acid for the prevention and treatment of cancer and HIV infection. Med Res Rev 2004;24:90–114. 14595673 10.1002/med.10053

[79] Chakraborty B, Dutta D, Mukherjee S, et al. Synthesis and biological evaluation of a novel betulinic acid derivative as an inducer of apoptosis in human colon carcinoma cells (HT-29). Eur J Med Chem 2015;102:93–105. 26248310 10.1016/j.ejmech.2015.07.035

[80] Chinthala Y, Manjulatha K, Sharma P, et al. Synthesis and cytotoxicity evaluation of novel andrographolide-1,2,3-triazole derivatives. J Heterocyclic Chem 2016;53:1902–10.

[81] Khan I, Guru SK, Rath SK, et al. A novel triazole derivative of betulinic acid induces extrinsic and intrinsic apoptosis in human leukemia HL-60 cells. Eur J Med Chem 2016;108:104–16. 26629862 10.1016/j.ejmech.2015.11.018

[82] Shi W, Tang N, Yan WD. Synthesis and cytotoxicity of triterpenoids derived from betulin and betulinic acid via click chemistry. J Asian Nat Prod Res 2015;17:159–69. 25379995 10.1080/10286020.2014.979164

[83] Djerassi C, Rosenkranz G, Pataki J, et al. Steroids, XXVII. Synthesis of allopregnane-3beta, 11beta, 17alpha-, 20beta, 21-pentol from cortisone and diosgenin. J Biol Chem 1952;194:115–8. 14927598

[84] Masood Ur R, Mohammad Y, Fazili KM, et al. Synthesis and biological evaluation of novel 3-O-tethered triazoles of diosgenin as potent antiproliferative agents. Steroids 2017;118:1–8. 27864018 10.1016/j.steroids.2016.11.003

[85] Mistry B, Patel RV, Keum YS, et al. Synthesis of Mannich base derivatives of berberine and evaluation of their anticancer and antioxidant effects. J Chem Res 2016;40:73–7.

[86] Wang J, Yang T, Chen H, et al. The synthesis and antistaphylococcal activity of 9, 13-disubstituted berberine derivatives. Eur J Med Chem 2017;127:424–33. 28092858 10.1016/j.ejmech.2017.01.012

[87] Jin X, Yan L, Li HJ, et al. Novel triazolyl berberine derivatives prepared via CuAAC click chemistry: synthesis, anticancer activity and structure-activity relationships. Anticancer Agents Med Chem 2015;15:89–98. 25482720

[88] Pavan Kumar P, Siva B, Venkateswara Rao B, et al. Synthesis and biological evaluation of bergenin-1,2,3-triazole hybrids as novel class of anti-mitotic agents. Bioorg Chem 2019;91:103161. 31387060 10.1016/j.bioorg.2019.103161

[89] Yang YX, Yan JW, Yan FL, et al. Synthesis and anti-tumour activity evaluation of bergenin derivatives. J Chem Res 2015;39:590–3.

[90] Sinha S, Kumaran AP, Mishra D, Paira P. Synthesis and cytotoxicity study of novel 3-(triazolyl)coumarins based fluorescent scaffolds. Bioorg Med Chem Lett 2016;26:5557–61. 27769619 10.1016/j.bmcl.2016.09.078

[91] Liu CF, Shen QK, Li JJ, et al. Synthesis and biological evaluation of novel 7-hydroxy-4-phenylchromen-2-one-linked to triazole moieties as potent cytotoxic agents. J Enzyme Inhib Med Chem 2017;32:1111–9. 28791908 10.1080/14756366.2017.1344982 PMC6445224

[92] Singh H, Singh JV, Gupta MK, et al. Triazole tethered isatin-coumarin based molecular hybrids as novel antitubulin agents: design, synthesis, biological investigation and docking studies. Bioorg Med Chem Lett 2017;27:3974–9. 28797799 10.1016/j.bmcl.2017.07.069

[93] Singh H, Kumar M, Nepali K, et al. Triazole tethered C5-curcuminoid-coumarin based molecular hybrids as novel antitubulin agents: design, synthesis, biological investigation and docking studies. Eur J Med Chem 2016;116:102–15. 27060762 10.1016/j.ejmech.2016.03.050

[94] S. El-Feraly F. Melampolides from *Magnolia grandiflora*. Phytochemistry 1984;23:2372–4.

[95] Janganati V, Ponder J, Balasubramaniam M, et al. MMB triazole analogs are potent NF-κB inhibitors and anti-cancer agents against both hematological and solid tumor cells. Eur J Med Chem 2018;157:562–81. 30121494 10.1016/j.ejmech.2018.08.010 PMC6281399

[96] Huang X, Shen QK, Zhang HJ, Li JL, et al. Design and synthesis of novel dehydroepiandrosterone analogues as potent antiproliferative agents. Molecules 2018;23:2243. 10.3390/molecules23092243 PMC6225165 30177642

[97] Yu B, Qi PP, Shi XJ, et al. Efficient synthesis of new antiproliferative steroidal hybrids using the molecular hybridization approach. Eur J Med Chem 2016;117:241–55. 27105028 10.1016/j.ejmech.2016.04.024

[98] Luan T, Cao LH, Deng H, et al. Design and synthesis of C-19 isosteviol derivatives as potent and highly selective antiproliferative agents. Molecules 2018;24:121. 10.3390/molecules24010121 PMC6337650 30598028

[99] Shen QK, Deng H, Wang SB, et al. Synthesis, and evaluation of in vitro and in vivo anticancer activity of 14-substituted oridonin analogs: a novel and potent cell cycle arrest and apoptosis inducer through the p53-MDM2 pathway. Eur J Med Chem 2019;173:15–31. 30981113 10.1016/j.ejmech.2019.04.005

[100] Aneja B, Queen A, Khan P, et al. Design, synthesis & biological evaluation of ferulic acid-based small molecule inhibitors against tumor-associated carbonic anhydrase IX. Bioorg Med Chem 2020;28:115424. 32209296 10.1016/j.bmc.2020.115424

[101] Ashour HF, Abou-Zeid LA, El-Sayed MA, Selim KB. 1,2,3-Triazole-chalcone hybrids: synthesis, in vitro cytotoxic activity and mechanistic investigation of apoptosis induction in multiple myeloma RPMI-8226. Eur J Med Chem 2020;189:112062. 31986406 10.1016/j.ejmech.2020.112062

[102] Yadav P, Lal K, Kumar A, et al. Green synthesis and anticancer potential of chalcone linked-1,2,3-triazoles. Eur J Med Chem 2017;126:944–53. 28011424 10.1016/j.ejmech.2016.11.030

[103] Reddy VG, Bonam SR, Reddy TS, et al. 4beta-amidotriazole linked podophyllotoxin congeners: DNA topoisomerase-IIalpha inhibition and potential anticancer agents for prostate cancer. Eur J Med Chem 2018;144:595–611. 29289884 10.1016/j.ejmech.2017.12.050

[104] Patra AK, Saxena J. A new perspective on the use of plant secondary metabolites to inhibit methanogenesis in the rumen. Phytochemistry 2010;71:1198–222. 20570294 10.1016/j.phytochem.2010.05.010

[105] Taia A, Essaber M, Oubella A, et al. Synthesis, characterization, and biological evaluation of new heterocyclic systems 1, 2, 3-triazole-isoxazoline from eugenol by the mixed condensation reactions. Synth Commun 2020;50:2052–65.

[106] Rodriguez-Hernandez D, Demuner AJ, Barbosa LC, et al. Novel hederagenin-triazolyl derivatives as potential anti-cancer agents. Eur J Med Chem 2016;115:257–67. 27017553 10.1016/j.ejmech.2016.03.018

[107] Phillips DR, Rasbery JM, Bartel B, Matsuda SP. Biosynthetic diversity in plant triterpene cyclization. Curr Opin Plant Biol 2006;9:305–14. 16581287 10.1016/j.pbi.2006.03.004

[108] Wei GF, Luan WJ, Wang S, et al. A library of 1,2,3-triazole-substituted oleanolic acid derivatives as anticancer agents: design, synthesis, and biological evaluation. Org Biomol Chem 2015;13:1507–14. 25476168 10.1039/c4ob01605j

[109] Guo S, Duan JA, Tang YP, et al. Characterization of triterpenic acids in fruits of *Ziziphus* species by HPLC-ELSD-MS. J Agric Food Chem 2010;58:6285–9. 20426471 10.1021/jf101022p

[110] Chouaib K, Delemasure S, Dutartre P, Jannet HB. Microwave-assisted synthesis, anti-inflammatory and anti-proliferative activities of new maslinic acid derivatives bearing 1,5- and 1,4-disubstituted triazoles. J Enzyme Inhib Med Chem 2016;31:130–47. 27435116 10.1080/14756366.2016.1193733

[111] Hua SX, Huang RZ, Ye MY, et al. Design, synthesis and in vitro evaluation of novel ursolic acid derivatives as potential anticancer agents. Eur J Med Chem 2015;95:435–52. 25841199 10.1016/j.ejmech.2015.03.051

[112] Wei ZY, Chi KQ, Wang KS, et al. Design, synthesis, evaluation, and molecular docking of ursolic acid derivatives containing a nitrogen heterocycle as anti-inflammatory agents. Bioorg Med Chem Lett 2018;28:1797–803. 29678461 10.1016/j.bmcl.2018.04.021

[113] Pang L, Liu CY, Gong GH, Quan ZS. Synthesis, in vitro and in vivo biological evaluation of novel lappaconitine derivatives as potential anti-inflammatory agents. Acta Pharm Sin B 2020;10:628–45. 32322467 10.1016/j.apsb.2019.09.002 PMC7161710

[114] Boshra AN, Abdu-Allah HHM, Mohammed AF, Hayallah AM. Click chemistry synthesis, biological evaluation and docking study of some novel 2'-hydroxychalcone-triazole hybrids as potent anti-inflammatory agents. Bioorg Chem 2020;95:103505 31901755 10.1016/j.bioorg.2019.103505

[115] Lipeeva AV, Dolgikh MP, Tolstikova TG, Shults EE. A study of plant coumarins. 18. Conjugates of coumarins with lupane triterpenoids and 1,2,3-triazoles: synthesis and anti-inflammatory activity. Russ J Bioorg Chem 2020;46:125–32.

[116] Ali Y, Alam MS, Hamid H, et al. Design, synthesis and biological evaluation of piperic acid triazolyl derivatives as potent anti-inflammatory agents. Eur J Med Chem 2015;92:490–500. 25596479 10.1016/j.ejmech.2015.01.001

[117] Aneja B, Azam M, Alam S, et al. Natural product-based 1,2,3-triazole/sulfonate analogues as potential chemotherapeutic agents for bacterial infections. ACS Omega 2018;3:6912–30. 30023966 10.1021/acsomega.8b00582 PMC6044994

[118] Hou W, Zhang G, Luo Z, et al. Identification of a diverse synthetic abietane diterpenoid library and insight into the structure-activity relationships for antibacterial activity. Bioorg Med Chem Lett 2017;27:5382–6. 29153424 10.1016/j.bmcl.2017.11.014

[119] Thibessard A, Haas D, Gerbaud C, et al. Complete genome sequence of *Streptomyces ambofaciens* ATCC 23877, the spiramycin producer. J Biotechnol 2015;214:117–8. 26410452 10.1016/j.jbiotec.2015.09.020

[120] Przybylski P. Modifications and biological activity of natural and semisynthetic 16-membered macrolide antibiotics. Curr Org Chem 2011;15:328–74.

[121] Klich K, Pyta K, Kubicka MM, et al. Synthesis, antibacterial, and anticancer evaluation of novel spiramycin-like conjugates containing C(5) triazole arm. J Med Chem 2016;59:7963–73. 27501415 10.1021/acs.jmedchem.6b00764

[122] Ingólfsdóttir K. Usnic acid. Phytochemistry 2002;61:729–36. 12453567 10.1016/s0031-9422(02)00383-7

[123] Yu X, Guo Q, Su G, et al. Usnic acid derivatives with cytotoxic and antifungal activities from the lichen *Usnea longissima*. J Nat Prod 2016;79:1373–80. 27186821 10.1021/acs.jnatprod.6b00109

[124] Bangalore PK, Vagolu SK, Bollikanda RK, et al. Usnic acid enaminone-coupled 1,2,3-triazoles as antibacterial and antitubercular agents. J Nat Prod 2020;83:26–35. 31858800 10.1021/acs.jnatprod.9b00475

[125] Sunitha V, Kumar AK, Jalapathi P, Lincoln CA. Synthesis and antimicrobial activity of bis-1,2,3-triazole based chalcones. Russ J Gen Chem 2020;90:154–9.

[126] Tang KW, Yang SC, Tseng CH. Design, synthesis, and anti-bacterial evaluation of triazolyl-pterostilbene derivatives. Int J Mol Sci 2019;20:4564. 10.3390/ijms20184564 PMC6769857 31540106

[127] Lal K, Yadav P, Kumar A, Kumar A, et al. Design, synthesis, characterization, antimicrobial evaluation and molecular modeling studies of some dehydroacetic acid-chalcone-1,2,3-triazole hybrids. Bioorg Chem 2018;77:236–44. 29421698 10.1016/j.bioorg.2018.01.016

[128] Anand A, Kulkarni MV, Joshi SD, Dixit SR. One pot click chemistry: a three component reaction for the synthesis of 2-mercaptobenzimidazole linked coumarinyl triazoles as anti-tubercular agents. Bioorg Med Chem Lett 2016;26:4709–13. 27595420 10.1016/j.bmcl.2016.08.045

[129] Lipeeva AV, Zakharov DO, Burova LG, et al. Design, synthesis and antibacterial activity of coumarin-1,2,3-triazole hybrids obtained from natural furocoumarin peucedanin. Molecules 2019;24:2126. 10.3390/molecules24112126 PMC6600338 31195697

[130] Tian X, Ruan J, Huang J, et al. Gossypol: phytoalexin of cotton. Sci China Life Sci 2016;59:122–9. 26803304 10.1007/s11427-016-5003-z

[131] Pyta K, Blecha M, Janas A, et al. Synthesis, structure and antimicrobial evaluation of a new gossypol triazole conjugates functionalized with aliphatic chains and benzyloxy groups. Bioorg Med Chem Lett 2016;26:4322–6. 27469129 10.1016/j.bmcl.2016.07.033

[132] Pertino MW, Theoduloz C, Butassi E, et al. Synthesis, antiproliferative and antifungal activities of 1,2,3-triazole-substituted carnosic acid and carnosol derivatives. Molecules 2015;20:8666–86. 26007173 10.3390/molecules20058666 PMC6272684

[133] Savanur HM, Naik KN, Ganapathi SM, et al. Click chemistry inspired design, synthesis and molecular docking studies of coumarin, quinolinone linked 1,2,3-triazoles as promising anti-microbial agents. Chemistryselect 2018;3:5296–303.

[134] Yadav P, Lal K, Kumar L, et al. Synthesis, crystal structure and antimicrobial potential of some fluorinated chalcone-1,2,3-triazole conjugates. Eur J Med Chem 2018;155:263–74. 29890388 10.1016/j.ejmech.2018.05.055

[135] Teixeira RR, Gazolla PAR, da Silva AM, et al. Synthesis and leishmanicidal activity of eugenol derivatives bearing 1,2,3-triazole functionalities. Eur J Med Chem 2018;146:274–86. 29407957 10.1016/j.ejmech.2018.01.046

[136] Rodriguez-Hernandez D, Barbosa LCA, Demuner AJ, et al. Highly potent anti-leishmanial derivatives of hederagenin, a triperpenoid from *Sapindus saponaria* L. Eur J Med Chem 2016;124:153–9. 27569196 10.1016/j.ejmech.2016.08.030

[137] Rodriguez-Hernandez D, Barbosa LCA, Demuner AJ, et al. Leishmanicidal and cytotoxic activity of hederagenin-bistriazolyl derivatives. Eur J Med Chem 2017;140:624–35. 29024910 10.1016/j.ejmech.2017.09.045

[138] Croft S. Antimalarial chemotherapy: mechanisms of action, resistance and new directions in drug discovery. Drug Discov Today 2001;6:1151. 11700215 10.1016/s1359-6446(01)02035-9

[139] Faidallah HM, Panda SS, Serrano JC, et al. Synthesis, antimalarial properties and 2D-QSAR studies of novel triazole-quinine conjugates. Bioorg Med Chem 2016;24:3527–39. 27298002 10.1016/j.bmc.2016.05.060

[140] Yadav N, Agarwal D, Kumar S, et al. In vitro antiplasmodial efficacy of synthetic coumarin-triazole analogs. Eur J Med Chem 2018;145:735–45. 29366931 10.1016/j.ejmech.2018.01.017

[141] Batista R, Garcia PA, Castro MA, et al. Synthesis, cytotoxicity and antiplasmodial activity of novel ent-kaurane derivatives. Eur J Med Chem 2013;62:168–76. 23353738 10.1016/j.ejmech.2012.12.010

[142] Santos JO, Pereira GR, Brandão GC, et al. Synthesis, in vitro antimalarial activity and in silico studies of hybrid kauranoid 1,2,3-triazoles derived from naturally occurring diterpenes. J Braz Chem Soc 2015;27:551–65.

[143] Kant R, Kumar D, Agarwal D, et al. Synthesis of newer 1,2,3-triazole linked chalcone and flavone hybrid compounds and evaluation of their antimicrobial and cytotoxic activities. Eur J Med Chem 2016;113:34–49. 26922227 10.1016/j.ejmech.2016.02.041

[144] Kumar S, Saini A, Gut J, et al. 4-Aminoquinoline-chalcone/-N-acetylpyrazoline conjugates: synthesis and antiplasmodial evaluation. Eur J Med Chem 2017;138:993–1001. 28756265 10.1016/j.ejmech.2017.07.041

[145] Zhang HB, Shen QK, Wang H, et al. Synthesis and evaluation of novel arctigenin derivatives as potential anti-*Toxoplasma gondii* agents. Eur J Med Chem 2018;158:414–27. 30237124 10.1016/j.ejmech.2018.08.087

[146] Luan T, Jin C, Jin CM, et al. Synthesis and biological evaluation of ursolic acid derivatives bearing triazole moieties as potential anti-*Toxoplasma gondii* agents. J Enzyme Inhib Med Chem 2019;34:761–72. 30836795 10.1080/14756366.2019.1584622 PMC6407578

[147] Guo HY, Jin C, Zhang HM, et al. Synthesis and biological evaluation of (+)-usnic acid derivatives as potential anti-*Toxoplasma gondii* agents. J Agric Food Chem 2019;67:9630–42. 31365255 10.1021/acs.jafc.9b02173

[148] de Souza TB, Caldas IS, Paula FR, et al. Synthesis, activity, and molecular modeling studies of 1,2,3-triazole derivatives from natural phenylpropanoids as new trypanocidal agents. Chem Biol Drug Des 2020;95:124–9. 31569301 10.1111/cbdd.13628

[149] Sahu A, Agrawal RK, Pandey R. Synthesis and systemic toxicity assessment of quinine-triazole scaffold with antiprotozoal potency. Bioorg Chem 2019;88:102939. 31028993 10.1016/j.bioorg.2019.102939

[150] Cassamale TB, Costa EC, Carvalho DB, et al. Synthesis and antitrypanosomastid activity of 1,4-diaryl-1,2,3-triazole analogues of neolignans veraguensin, grandisin and machilin G. J Braz Chem Soc 2016;27:1217–28.

[151] Artyushin OI, Moiseeva AA, Zarubaev VV, et al. Synthesis of camphecene and cytisine conjugates using click chemistry methodology and study of their antiviral activity. Chem Biodivers 2019;16:e1900340. 31647170 10.1002/cbdv.201900340

[152] Zhang C, Li N, Niu F. Baicalein triazole prevents respiratory tract infection by RSV through suppression of oxidative damage. Microb Pathog 2019;131:227–33. 30943433 10.1016/j.micpath.2019.03.026

[153] Harej A, Macan AM, Stepanic V, et al. The antioxidant and antiproliferative activities of 1,2,3-triazolyl-L-ascorbic acid derivatives. Int J Mol Sci 2019;20:4735. 10.3390/ijms20194735 PMC6801448 31554245

[154] Dharavath R, Nagaraju N, Reddy MR, et al. Microwave-assisted synthesis, biological evaluation and molecular docking studies of new coumarin-based 1,2,3-triazoles. RSC Advances 2020;10:11615–23. 10.1039/d0ra01052a PMC9050871 35496603

[155] Wang W, Wang W, Yao G, et al. Novel sarsasapogenin-triazolyl hybrids as potential anti-Alzheimer’s agents: design, synthesis and biological evaluation. Eur J Med Chem 2018;151:351–62. 29635167 10.1016/j.ejmech.2018.03.082

[156] Torres FC, Goncalves GA, Vanzolini KL, et al. Combining the pharmacophore features of coumarins and 1,4-substituted 1,2,3-triazoles to design new acetylcholinesterase inhibitors: fast and easy generation of 4-methylcoumarins/1,2,3-triazoles conjugates via click chemistry. J Braz Chem Soc 2016;27:1541–50.

[157] Park JY, Shin S, Park KC, et al. Synthesis and in vitro assay of new triazole linked decursinol derivatives showing inhibitory activity against cholinesterase for Alzheimer’s disease therapeutics. J Korean Chem Soc 2016;60:125–30.

[158] Montanari S, Scalvini L, Bartolini M, et al. Fatty acid amide hydrolase (FAAH), acetylcholinesterase (AChE), and butyrylcholinesterase (BuChE): networked targets for the development of carbamates as potential anti-Alzheimer’s disease agents. J Med Chem 2016;59:6387–406. 27309570 10.1021/acs.jmedchem.6b00609

[159] Saeedi M, Safavi M, Karimpour-Razkenari E, et al. Synthesis of novel chromenones linked to 1,2,3-triazole ring system: investigation of biological activities against Alzheimer’s disease. Bioorg Chem 2017;70:86–93. 27914694 10.1016/j.bioorg.2016.11.011

[160] Iraji A, Firuzi O, Khoshneviszadeh M, et al. Multifunctional iminochromene-2H-carboxamide derivatives containing different aminomethylene triazole with BACE1 inhibitory, neuroprotective and metal chelating properties targeting Alzheimer’s disease. Eur J Med Chem 2017;141:690–702. 29107423 10.1016/j.ejmech.2017.09.057

[161] Chekir S, Debbabi M, Regazzetti A, et al. Design, synthesis and biological evaluation of novel 1,2,3-triazole linked coumarinopyrazole conjugates as potent anticholinesterase, anti-5-lipoxygenase, anti-tyrosinase and anti-cancer agents. Bioorg Chem 2018;80:189–94. 29940340 10.1016/j.bioorg.2018.06.005

[162] Jalili-Baleh L, Forootanfar H, Kucukkilinc TT, et al. Design, synthesis and evaluation of novel multi-target-directed ligands for treatment of Alzheimer’s disease based on coumarin and lipoic acid scaffolds. Eur J Med Chem 2018;152:600–14. 29763808 10.1016/j.ejmech.2018.04.058

[163] Moradi A, Faraji L, Nadri H, et al. Synthesis, docking study, and biological evaluation of novel umbellipherone/hymecromone derivatives as acetylcholinesterase/butyrylcholinesterase inhibitors. Med Chem Res 2018;27:1741–7.

[164] Rastegari A, Nadri H, Mahdavi M, et al. Design, synthesis and anti-Alzheimer’s activity of novel 1,2,3-triazole-chromenone carboxamide derivatives. Bioorg Chem 2019;83:391–401. 30412794 10.1016/j.bioorg.2018.10.065

[165] Sheeja ADB, Nair MS. Facile isolation of (E)-labda-8(17),12-diene-15,16-dial from *Curcuma amada* and its conversion to other biologically active compounds. Indian J Chem B 2014;53:319–24.

[166] Jalaja R, Leela SG, Valmiki PK, et al. Discovery of natural product derived labdane appended triazoles as potent pancreatic lipase inhibitors. ACS Med Chem Lett 2018;9:662–6. 30034597 10.1021/acsmedchemlett.8b00109 PMC6047173

[167] Kahveci B, Yılmaz F, Menteşe E, Ülker S. Design, synthesis, and biological evaluation of coumarin-triazole hybrid molecules as potential antitumor and pancreatic lipase agents. Arch Pharm (Weinheim) 2017;350:e1600369. 10.1002/ardp.201600369 28543820

[168] Zhang TJ, Li SY, Yuan WY, et al. Discovery and biological evaluation of some (1H-1,2,3-triazol-4-yl)methoxybenzaldehyde derivatives containing an anthraquinone moiety as potent xanthine oxidase inhibitors. Bioorg Med Chem Lett 2017;27:729–32. 28131711 10.1016/j.bmcl.2017.01.049

